# Childhood and adult socioeconomic status influence on late-life healthy longevity: evidence from the Chinese longitudinal healthy longevity survey

**DOI:** 10.3389/fpubh.2024.1352937

**Published:** 2024-09-30

**Authors:** Yuanyan Chen

**Affiliations:** School of Public Finance and Taxation, Capital University of Economics and Business, Beijing, China

**Keywords:** healthy longevity, childhood socioeconomic status, adult socioeconomic status, life course perspective, multi-state models, frailty index

## Abstract

**Background:**

Older people in low- and middle-income countries are more susceptible to the impact of childhood experiences. This study comprehensively examines how childhood socioeconomic status (SES) and adult SES collectively influence late-life healthy longevity from a life course perspective, providing insights for shaping health-related policies.

**Methods:**

This study analyzed data from the Chinese Longitudinal Healthy Longevity Survey (1998–2018) with 37,264 individuals aged 65 and above. Using R software, we applied continuous-time multi-state models incorporating the Rockwood frailty index with 38 indicators to assess participants’ health. Childhood SES or life course SES trajectories were core explanatory variables, while age and gender were controlled. Multinomial regression estimated annual transition probabilities between different states, and the multi-state life table method calculated total and frailty-specific life expectancy (LE).

**Results:**

(1) Social mobility among older people in China showed an upward trend from childhood to adulthood. (2) Transition probabilities for robust-frailty, robust-dead, and frailty-dead increased with age, while frailty-robust decreased. Transition probabilities and LE varied across different childhood SES (low, medium, high) or life-course SES trajectory categories (low-low, low-medium, low-high, medium-low, medium-medium, medium-high, high-low, high-medium, high-high), with probabilities of robust-frailty, robust-dead, and frailty-dead decreasing sequentially across different categories, and frailty-robust increasing sequentially across different categories. Total LE, robust LE, and robust LE proportion increased sequentially across different categories, while frailty LE decreased sequentially across different categories. (3) Women had higher total LE and frailty incidence, but lower recovery rate, mortality risk, robust LE, and robust LE proportion compared to men.

**Conclusion:**

Favorable childhood SES and lifelong accumulation of SES advantages protect against frailty morbidity, improve recovery rate, reduce mortality risk, and increase total LE, robust LE, and robust LE proportion. High childhood SES has a stronger protective effect than high adult SES, indicating the lasting impact of childhood conditions on healthy longevity. Systematic interventions in education, food supply, and medical accessibility for children from impoverished families are crucial.

## 1 Introduction

According to the findings of the China Seventh National Population Census, the population aged 65 and over accounted for 13.50 percent of the total national population, totaling 190 million individuals (1). Furthermore, it is noteworthy that China accounted for 25% of the global population aged 65 and over during the same period (2). In terms of life expectancy (LE), there has been a notable increase in both male and female LE at birth in China. From 1978 to 2021, male LE at birth rose from 61.1 years to 75.5 years, while female LE at birth increased from 65.5 years to 81.2 years (2). In contemporary China, a significant proportion of the older population has lived through challenging circumstances during their formative years, including war and social instability (3). Nevertheless, the majority of older people in China have experienced substantial enhancements in their living conditions throughout their adult and senior years (4). The contrast between the circumstances of their youth and the quality of life in their later years could exert a significant influence on their well-being (4–6). Therefore, applying a life course perspective to investigate health concerns among older people in China holds distinctive significance. Furthermore, China’s substantial population, accelerated aging, and rapid social and economic progress during the last 45 years have established the nation as a unique and globally influential research environment.

The life course approach suggests that the development of age-related illnesses, physical impairments, and mortality is influenced by a range of factors across different life phases, with socioeconomic status (SES) assuming a central role (7). The life course perspective encompasses the critical period model, the pathway model, the social trajectory model, the accumulation model, and the social mobility model (8, 9). The critical period model posits that adverse childhood experiences, once embedded in biological systems, may exert long-lasting influences on adult health, irrespective of later life exposures (10). Multiple studies in the United States, Sweden, and Finland have corroborated the critical period model (11–15). The pathway model underscores the impact of early socioeconomic conditions on adult SES and their influence on life course trajectories (16), as confirmed by several studies in the United States and Europe (14, 17, 18). The social trajectory model delineates the interconnected pathways of risk, wherein one adverse exposure amplifies the likelihood of subsequent negative exposures (9, 19). The accumulation model emphasizes the impact of progressively accumulating risk factors over the course of one’s life on shaping health outcomes (20, 21), as evidenced by several studies in Sweden, Finland, the UK, France, and the USA (9, 11, 13, 15, 22–27). The model of social mobility argues that SES attained in adulthood has the potential to ameliorate or counteract the impacts of early-life circumstances (8, 28), as confirmed by several studies in France, Sweden, Finland, and the United States (11–15, 22, 23, 25).

Research on health in old age from a life course perspective has primarily focused on high-income countries (HICs), with limited attention to low- and middle-income countries (LMICs), although some evidence exists from China. An investigation conducted through the Chinese Longitudinal Healthy Longevity Survey (CLHLS) in 2002 and 2005 revealed a potential direct correlation between childhood SES and cognitive, physical, and self-rated health, as well as mortality risk, while indicating a limited influence of adult SES on these associations. Examination of social mobility patterns demonstrated that maintaining a high SES consistently offered the greatest protection against cognitive decline and mortality risk, whereas a sustained low SES had the most detrimental impact. In terms of self-rated health, it was observed that only a sustained high SES provided substantial protection, with upward mobility failing to counteract the negative effects of lower childhood SES on self-rated health in later life (29). A separate investigation, utilizing data from the CLHLS spanning 1998, 2000, 2002, and 2005, revealed that women with non-agricultural working fathers exhibited more advantageous developmental paths (30). Study showed a correlation between SES during childhood and adulthood and the development of functional disability in older age. Specifically, lower paternal education was associated with limitations in instrumental activities of daily living (IADL). Conversely, higher educational attainment and improved economic circumstances in adulthood were identified as protective factors against both activities of daily living (ADL) and IADL disabilities (31). Another study found that childhood SES directly affects type 2 diabetes and indirectly influences it through adult SES (32). Additionally, a study revealed that low childhood SES has an unfavorable indirect effect on oral health mediated through adult SES (33).

The literature on health among older people from a life course perspective typically uses indicators such as self-rated health, ADL, IADL, and functional limitations (FL) to assess health status (13, 22, 29, 31). However, these indicators may not be comprehensive enough. Frailty, a common geriatric syndrome associated with disability, mortality, and hospitalization, is receiving increasing attention (34). Frailty is typically assessed using the frailty index (FI) (35). Research in Europe has shown that immigrants born in LMICs exhibit higher levels of frailty in Nordic/Western Europe (36). Multiple UK studies have explored frailty. One study revealed that lower childhood SES was associated with higher FI (37). Additionally, a study on twins showed a negative correlation between father’s occupational class and the participants’ Rockwood FI (38), while participants’ educational levels mediated the relationship between early life factors and FI (38, 39). A study in Germany found that women and those living below the poverty line have higher levels of FI in their later years (40). Furthermore, there is limited evidence from LMICs. A cross-border survey of older people from five major cities in Latin America revealed that childhood and adulthood adversity are associated with higher rates of frailty in later years, with women exhibiting a higher prevalence of frailty than men (41). There are only two pieces of evidence from China. A recent investigation revealed that the FI experienced an average increase of 0.10 within the most disadvantaged subpopulation compared to the most advantaged subpopulation (42). A comparative study between China and Europe found that experiencing three or more childhood adversities is associated with an increase in FI levels (43).

The life course theory, while widely supported, presents conflicting perspectives. An investigation into the health outcomes of U.S. men unveiled that the association between childhood SES and adult health exhibited a reduced strength among men from racial and ethnic minority groups in comparison to White men. Furthermore, the impact of childhood SES on health appeared to diminish with age specifically for African American and Mexican American men (44). In Sweden, a study found that gap in self-rated health increases from early to middle adulthood, but remains unchanged after the age of 55 (45). Research in China indicated that health inequalities induced by childhood SES gradually widen before 60 years of age, but then gradually converge after 60 years of age (46), aligning with the age-neutral effect theory (47, 48).

Older people in LMICs are more influenced by childhood experiences compared to HICs (32, 49). However, research on LMICs is limited and the conclusions are inconsistent. Only a small number of studies on healthy aging in China from a life course perspective use the FI to measure health diversity among older people (42, 43). Several studies have used various models such as linear regression (38, 42, 43), linear mixed effects (26, 46), structural equation (17, 18, 32, 33, 40), logistic regression (9, 13, 23–25, 29, 31, 41), and growth curve models (14, 44). Nevertheless, the dynamics of disability incidence, recovery, and mortality interaction might be neglected by these models. Continuous-time multi-state models offer a comprehensive insight into the life experiences of disability and mitigate the risk of biased disability level estimates at the population scale (4, 50). There is scarce literature utilizing continuous time multi-state models to study healthy longevity in later years from a life course perspective. Only two exceptions exist, with one study using relatively short-term data and a limited sample size (4). Another study explored the impact of childhood starvation on healthy longevity in later years, using relatively limited indicators for childhood and adult SES, and a single measure for late health status (51).

This study aims to contribute to the existing body of research by employing the Rockwood FI, which includes 38 indicators, to thoroughly examine the combined impact of childhood and adult SES on healthy longevity in later life. Utilizing data from eight waves of CLHLS spanning from 1998 to 2018, and encompassing a substantial sample size, this research seeks to answer the primary question: How do childhood and adult SES jointly influence healthy longevity among the older population in China? The objective is to provide a nuanced understanding of the long-term effects of SES across the life course on health outcomes in old age. This is achieved through the application of continuous-time multi-state models, which effectively capture the dynamics of disability incidence, recovery, and mortality. The findings aim to inform health-related policies and interventions by highlighting the critical role of childhood SES and the lifelong accumulation of SES advantages in shaping health trajectories in later years, with a particular emphasis on the greater impact of childhood SES.

## 2 Methods

### 2.1 Data

The study utilized information obtained from the Chinese Longitudinal Healthy Longevity Survey (CLHLS), which involved conducting eight repeated cross-sectional surveys spanning from 1998 to 2018. The survey interviewed individuals aged 65 and above in randomly selected counties and cities across 23 provinces/municipalities/autonomous regions in China. The initial and follow-up surveys, which included replacements for deceased participants, were conducted in a randomly selected half of the counties and cities within 22 of mainland China’s 31 provinces during the years 1998, 2000, 2002, and 2005. The provinces surveyed were Liaoning, Jilin, Heilongjiang, Hebei, Beijing, Tianjin, Shanxi, Shaanxi, Shanghai, Jiangsu, Zhejiang, Anhui, Fujian, Jiangxi, Shandong, Henan, Hubei, Hunan, Guangdong, Guangxi, Sichuan, and Chongqing. Hainan was added as the 23rd province in the 5th, 6th, and 7th waves. The regions covered by these surveys represent approximately 85 percent of China’s total population (52). The surveys included 631 counties and cities in 1998, 777 in 2000, and 866 in 2002, with all subsequent surveys from 2002 onwards consistently including over 860 counties and cities (53). The CLHLS survey stands out for its unparalleled breadth of older adult participants in longitudinal studies on a global scale (54), earning acclaim from scholars both domestically and abroad (55). The study received ethical approval from the Biomedical Ethics Committee of Peking University under the reference number IRB00001052-13,074, and written informed consent was obtained from all participants or their authorized representatives.

During the period from 1998 to 2014, a cohort of 44,298 participants was individually recruited and longitudinally monitored until 2018. After applying the criteria for the multi-state model to investigate frailty status transitions, the final analysis encompassed 37,264 participants, following the exclusion of individuals below 65 years of age, respondents to a single survey wave, or those lacking age or frailty index (FI) data. The achieved response rate stood at 84.12%. Comprehensive findings are delineated in Supplementary Figure S1.

### 2.2 Variables

#### 2.2.1 The frailty index (FI) and death

The Rockwood Frailty Index (FI) provides a dependable assessment of frailty in the CLHLS (54, 56, 57), employing standardized protocols (58) and incorporating 38 self-reported health deficits (59–61). These deficits, including ADL, bodily function, cognitive function, psychological status, sensory abilities, self-reported health, chronic illness, and other factors, were used to construct the FI, the detailed results are available in Supplementary Table S1, S2. Deficits with missing information were excluded from both the denominator and the numerator. In cases where over 30% of the deficits had missing data, the FI was classified as incomplete (61). The frailty index, graded on a scale from 0.00 to 1.00, reflects the individual’s health status, with higher scores signifying increased frailty. In line with prior study (62), the frailty index is stratified into two tiers: robust (frailty index score ≤ 0.10) and frailty (frailty index score > 0.10). Data regarding mortality is acquired from close relatives or primary caregivers, and the accuracy of the date and cause of death is confirmed through the examination of death certificates (63), thereby ensuring the high quality of the data. The follow-up duration is determined by measuring the time elapsed between the initial interview date and the date of death. During each survey wave, participants are categorized as either deceased or into one of two frailty states (robust and frailty), as depicted in Supplementary Figure S2. The health status of the respondents is represented by the variable state, with a value of 1 indicating robust, 2 indicating frailty, and 3 indicating deceased.

#### 2.2.2 Estimating childhood and adulthood SES

##### 2.2.2.1 Childhood SES

Drawing from existing literature, the key indicators for assessing childhood SES encompass parental education level (6, 14, 18, 32, 33, 44, 46, 64), father’s occupation (6, 14, 17, 18, 32, 33, 44, 46, 64), the presence of food scarcity or the economic status of childhood families (6, 14, 25, 33, 44, 64), whether the birthplace or primary childhood residence was in an urban setting (6, 33), and the availability of medical care (6, 33).

Childhood SES was assessed using four indicators: (1) birth type, indicating urban (1) or rural (0) birth; (2) hunger-free, denoting absence of frequent childhood hunger (1) or presence of hunger (0); (3) father’s occupation, categorized as non-agricultural (1) or agricultural/housework (0) based on specific job types (6, 31, 32); and (4) medical care, indicating adequate childhood medical service (1) or lack thereof (0). The four variables are well validated and commonly used in the literature on childhood SES for Chinese older people (6, 29, 31, 33). In the early 20th century, China was predominantly an agricultural society with low education levels (32, 65), resulting in approximately 95% of respondents having mothers without formal education. The CLHLS only started collecting data on the education level of respondents’ parents in 2005, hence parental education level indicators were not included in the selection of childhood SES indicators.

The missing values for the four indicators are as follows: birth type missing value accounts for only 0.238%, hunger-free missing value accounts for 2.938%, father’s occupation missing value accounts for only 0.606%, and the missing value for medical care accounts for 6.635%. Multiple imputation by chained equations was used to impute values for missing data (4). The imputation model for the childhood SES index included all other variables comprising the childhood SES index, and 100 imputed datasets were generated. Values for missing data were estimated as the mean of these 100 imputed values, rounded to the nearest whole number, and then converted to either 0 or 1. The scores of the four indicators for each respondent were summed, the total scores of the childhood SES index for all respondents were sorted, and the total scores were divided into 4 equal-width bins, categorizing childhood SES into three categories: low, medium, and high, denoted by 0, 1, 2, respectively (29).

##### 2.2.2.2 Adulthood SES

Drawing from existing literature, the key indicators for assessing adult SES encompass educational attainment (18, 25, 33, 44, 46, 66), household income and wealth (14, 17, 44, 46, 66), and occupation (17, 18). Furthermore, current urban–rural residence, pension status, and access to health care are endorsed by existing literature as effective proxies of SES in modern-day China (29, 67–69).

The assessment of adult SES encompasses six key indicators: (1) residence, reflecting the interviewee’s urban or rural dwelling, with urban areas assigned a value of 1 and rural areas a value of 0; (2) education, representing the years of schooling, with a value of 0 for no schooling and 1 for any years of schooling; (3) occupation, indicating the interviewee’s primary occupation before age 60, with non-agricultural jobs assigned a value of 1 and agricultural jobs or housework assigned a value of 0; (4) pension, denoting the presence of a pension, with a value of 1 for “yes” and 0 for “no”; (5) financial sufficiency, signifying whether the interviewee’s financial resources are adequate, with a value of 1 for “yes” and 0 for “no” or “so so”; (6) medical care at age 60, indicating whether the interviewee received adequate medical service, with a value of 1 for “yes” or “not sick” and 0 for “no.” Our analysis assumes the stability of adult SES over time due to the limitations of the multi-state life table model in accommodating time-varying covariates. Consequently, income was not included as a measure of adult SES, given its time-varying nature.

Regarding missing data, the residence indicator exhibited no missing values, while the missing value rates for education, occupation, pension, financial sufficiency, and medical care at age 60 were 0.429, 0.379, 0.106, 1.655, and 4.728%, respectively. To address missing data, multiple imputation by chained equations was employed, generating 100 imputed datasets (4). The imputation model for the adulthood SES index encompassed all other variables comprising the index. The imputed values were estimated as the mean of the 100 imputed values, rounded to the nearest whole number, and then converted to either 0 or 1. Subsequently, the scores for the six indicators for each interviewee were summed, and the summed scores for all interviewees’ adulthood SES index were categorized into three classes: low, medium, and high, represented by 0, 1, and 2, respectively (29).

##### 2.2.2.3 Life course SES trajectories

Life course SES trajectories are constructed based on a combination of childhood and adult SES indicators, resulting in nine distinct trajectories (3 childhood SES groups × 3 adulthood SES groups): low-low, low-medium, low-high, medium-low, medium-medium, medium-high, high-low, high-medium, high-high, denoted by 1, 2, 3, 4, 5, 6, 7, 8, 9, respectively (4). The age of the participants is represented by the variable “age.” Previous research in the field has extensively explored gender disparities in health among older people (15, 22, 40, 41, 66, 70). Gender is represented by the variable “gender,” with male coded as 1 and female as 0. In the covariate selection process, this study deliberately excluded variables that could be influenced by frailty, such as indicators of present health behaviors or living conditions. Factors like changes in health behaviors (e.g., smoking or physical activity) may be a response to changes in frailty status (30), and these variables are also prone to change over time. Time-varying covariates were not included.

### 2.3 Modeling stochastic health processes and life expectancy computation

Continuous-time multi-state models, based on the principles of continuous-time Markov chains, excel at illustrating random health-related developments over time, enabling the portrayal of different health phases or mortality. At a specific age, residual life expectancy (LE) can be divided into two categories: robust LE and frailty LE, with their collective sum representing the total LE. The multi-state life table (MSLT) method, widely endorsed in global literature, relies on cohort data to precisely depict the health status of the research population, resulting in more reliable research findings (4, 51, 61, 71, 72).

Initially, a comprehensive multi-state Markov model incorporating covariates was implemented utilizing the msm package within the R programming language (73). Following this, we utilized continuous-time multi-state models to calculate total and frailty-specific LE in a two-stage procedure. Initially, a multinomial regression was applied to estimate the annual probability of transitioning between different health states, producing transition probability matrices for each gender and SES (either childhood SES or life course SES trajectory) combination. Subsequently, the annual transition probabilities derived from the initial stage were fed into the MSLT method to compute total and frailty-specific LE (robust LE and frailty LE) using the elect package in the R programming language (74). For detailed explanations, please refer to Supplementary material [Section 3 Estimation methods of life expectancy (LE)].

In this study, the maximum age of the participants is 125 years old, and it was assumed that the maximum human age was 130 years old. When performing the calculation using the default “step” method for numerical approximation, the integral was approximated on a 0.1-year grid. A standard error was computed for each estimate using a bootstrapping method that involved 500 repeated estimates through random draws.

### 2.4 Statistical analysis

The analysis commenced with a descriptive statistical examination of the data, focusing on the distribution of participants’ age, gender, childhood SES, adulthood SES, and life course SES (child–adult). Subsequently, we present the annual transition probabilities based on childhood SES or life course SES trajectory, illustrating the transition probabilities from robust to frailty, frailty to robust, robust to dead, and frailty to dead by childhood SES or by life course SES trajectory. Finally, an analysis of life expectancy based on childhood SES or life course SES trajectory is presented, providing estimated robust, frailty, and total life expectancy (LE) at ages 65 and 75 by childhood SES or by life course SES trajectory.

## 3 Results

### 3.1 Descriptive analysis

In Table 1, the final analysis included 37,264 participants, with a mean age of 88.44 years (SD: 11.18). The proportion of males (41.2%) was slightly lower than that of females. Regarding childhood SES, 67.7% were classified as low, 20.7% as medium, and 11.6% as high. Notably, 98% of the participants were born between 1895 and 1945, a period marked by challenging living circumstances, societal upheaval, prolonged food scarcity, and childhood exposure to infectious diseases (3), leading to a relatively high proportion of participants with disadvantaged childhood SES. In terms of adulthood SES, 52.1% were classified as low, 33.5% as medium, and 14.4% as high, reflecting an overall upward social mobility trend from childhood to adulthood among older people in China. Specifically, based on social mobility classification (29, 64, 75), 41.6% of participants consistently remained in a low position (low–low) in terms of Life Course SES (child–adult), 8.2% consistently remained in a medium position (medium–medium), and 5.2% consistently remained in a high position (high–high). The proportion of participants experiencing upward mobility (low–medium, low–high, medium–high) was 29.7%, while the proportion experiencing downward mobility (medium–low, high–low, high–medium) was only 15.2%, significantly lower than the proportion experiencing upward mobility. This study categorized participants’ childhood SES and adulthood SES into three classes (low, medium, and high), represented by 0, 1, and 2, respectively. Therefore, according to the accumulation model (13, 20, 21), the proportion of participants with an accumulated advantage of 0 (low–low) was 41.6%, an accumulated advantage of 1 (low–medium, medium–low) was 29.3%, an accumulated advantage of 2 (low–high, medium–medium, high–low) was 15.4%, an accumulated advantage of 3 (medium–high, high–medium) was 8.4%, and an accumulated advantage of 4 (high–high) was 5.2%, showing a decreasing trend.

**Table 1:** Participant demographics at baseline, *n* (%).

		Enrolled in 1998	Enrolled in 2000	Enrolled in 2002	Enrolled in 2005	Enrolled in 2008	Enrolled in 2011	Enrolled in 2014	Total
*N*		8,090	5,383	8,362	5,941	7,547	1,071	870	37,264
Age (mean (SD))		92.33 (7.70)	90.37 (7.51)	83.06 (12.76)	88.02 (12.20)	89.26 (11.45)	87.94 (12.47)	88.48 (9.88)	88.44 (11.18)
Gender	Male	3,215 (39.7)	2,293 (42.6)	3,585 (42.9)	2,406 (40.5)	3,049 (40.4)	448 (41.8)	373 (42.9)	15,369 (41.2)
	Female	4,875 (60.3)	3,090 (57.4)	4,777 (57.1)	3,535 (59.5)	4,498 (59.6)	623 (58.2)	497 (57.1)	21,895 (58.8)
Childhood SES	Low	3,895 (48.2)	2,988 (55.5)	6,093 (72.9)	4,403 (74.1)	6,205 (82.2)	925 (86.4)	724 (83.2)	25,233 (67.7)
	Medium	2,713 (33.5)	1,522 (28.3)	1,412 (16.9)	928 (15.6)	898 (11.9)	119 (11.1)	113 (13.0)	7,705 (20.7)
	High	1,482 (18.3)	873 (16.2)	857 (10.3)	610 (10.3)	444 (5.9)	27 (2.5)	33 (3.8)	4,326 (11.6)
Adulthood SES	Low	4,555 (56.3)	2031 (37.7)	4,214 (50.4)	3,035 (51.1)	4,422 (58.6)	655 (61.2)	505 (58.1)	19,417 (52.1)
	Medium	2,666 (33.0)	2,343 (43.5)	2,697 (32.3)	1870 (31.5)	2,238 (29.7)	373 (34.8)	298 (34.3)	12,485 (33.5)
	High	869 (10.7)	1,009 (18.7)	1,451 (17.4)	1,036 (17.4)	887 (11.8)	43 (4.0)	67 (7.7)	5,362 (14.4)
Life course SES (child–adult)	Low-low	2,692 (33.3)	1,462 (27.2)	3,612 (43.2)	2,666 (44.9)	4,044 (53.6)	602 (56.2)	440 (50.6)	15,518 (41.6)
	Low-medium	994 (12.3)	1,214 (22.6)	1878 (22.5)	1,286 (21.7)	1731 (22.9)	292 (27.3)	250 (28.7)	7,645 (20.5)
	Low-high	209 (2.6)	312 (5.8)	603 (7.2)	451 (7.6)	430 (5.7)	31 (2.9)	34 (3.9)	2070 (5.6)
	Medium-low	1,538 (19.0)	484 (9.0)	513 (6.1)	306 (5.2)	337 (4.5)	48 (4.5)	63 (7.2)	3,289 (8.8)
	Medium-medium	940 (11.6)	750 (13.9)	559 (6.7)	368 (6.2)	351 (4.7)	63 (5.9)	38 (4.4)	3,069 (8.2)
	Medium-high	235 (2.9)	288 (5.4)	340 (4.1)	254 (4.3)	210 (2.8)	8 (0.8)	12 (1.4)	1,347 (3.6)
	High-low	325 (4.0)	85 (1.6)	89 (1.1)	63 (1.1)	41 (0.5)	5 (0.5)	2 (0.2)	610 (1.6)
	High-medium	732 (9.1)	379 (7.0)	260 (3.1)	216 (3.6)	156 (2.1)	18 (1.7)	10 (1.2)	1771 (4.8)
	High-high	425 (5.3)	409 (7.6)	508 (6.1)	331 (5.6)	247 (3.3)	4 (0.4)	21 (2.4)	1945 (5.2)

The differences in childhood SES (low, medium, high) were examined, revealing statistically significant differences among the three groups in terms of gender, age, and state (*p* < 0.001). Similarly, the analysis of adulthood SES (low, medium, high) and life course SES (low-low, low-medium, low-high, medium-low, medium-medium, medium-high, high-low, high-medium, high-high) demonstrated statistically significant differences in gender, age, and state (p < 0.001). Detailed results of the hypothesis tests are provided in Supplementary Table S3.

### 3.2 Annual transition probability

#### 3.2.1 Annual transition probability by childhood SES

**Figure 1:**
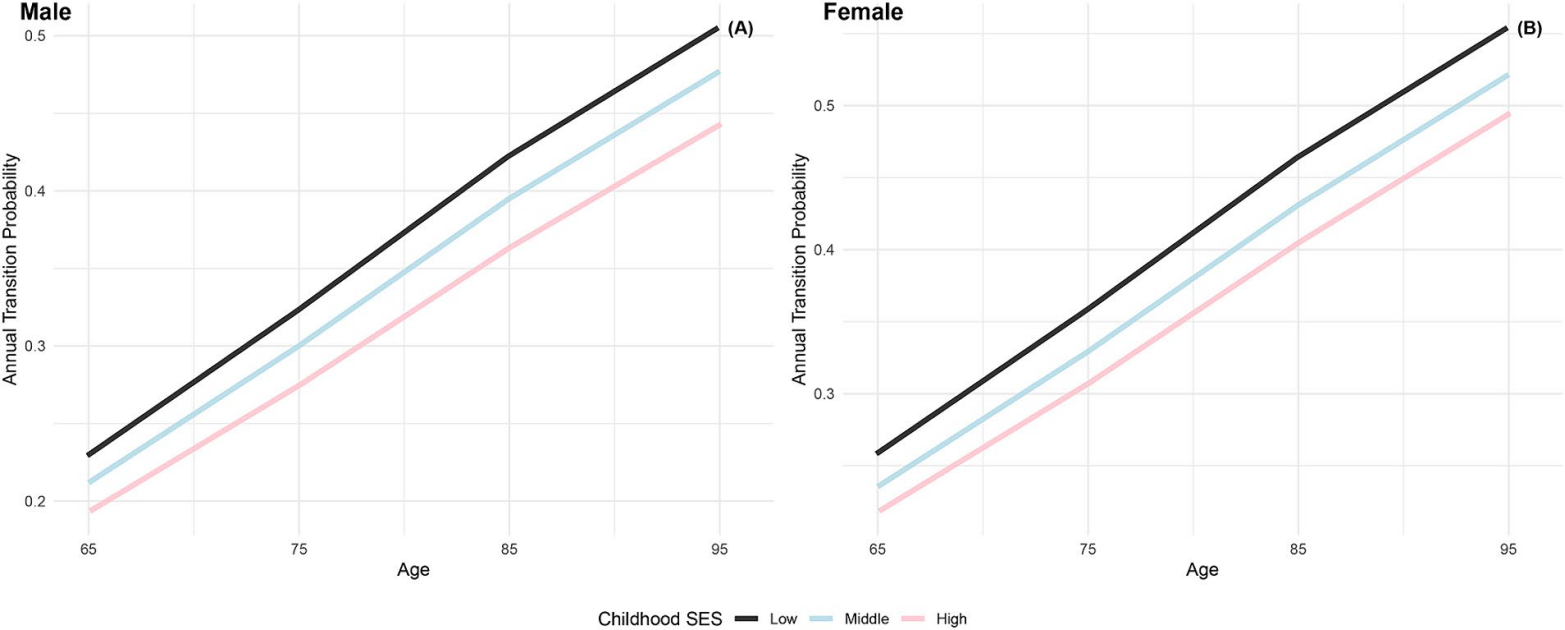
Annual transition probabilities from robust to frailty based on childhood SES, CLHLS 1998–2018. **(A)** Male. **(B)** Female.

Figure 1 depicts the annual transition probabilities from robust to frailty based on childhood SES, with separate analyses for males and females. The data indicates that, as individuals age, the annual transition probabilities from robust to frailty increase across all three categories of childhood SES (low, medium, high), regardless of gender. Specifically, male participants with low, medium, and high childhood SES show annual transition probabilities from robust to frailty ranging from 23 to 51%, 21 to 48%, and 19 to 44%, respectively, between the ages of 65 and 95. These results suggest that the annual transition probabilities from robust to frailty for male participants in the three categories of childhood SES (low, medium, high) have decreased sequentially, with the lowest transition probability for the most favorable childhood SES category, the highest for the least favorable, and the medium category falling in between, with female participants showing the same characteristics. Moreover, across all ages, female participants in all three childhood SES categories (low, medium, high) had higher annual transition probabilities from robust to frailty than their male counterparts in the same categories. These findings align with prior research in Latin American cities (41) and a study in Germany (40), which concluded that the frailty rate is higher in women than in men. Furthermore, this preliminary evidence supports the critical period model (10), suggesting that advantageous childhood SES exerts a protective influence on frailty rates, regardless of gender.

**Figure 2:**
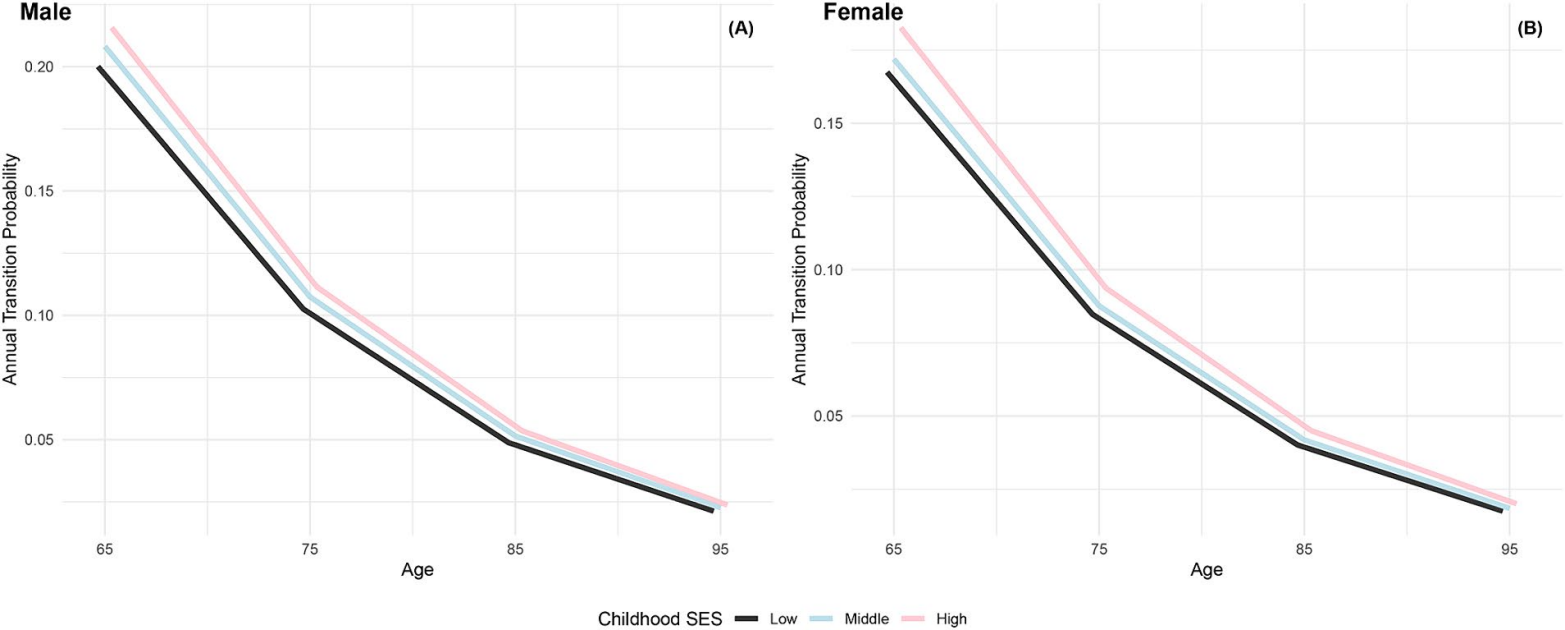
Annual transition probabilities from frailty to robust based on childhood SES, CLHLS 1998–2018. **(A)** Male. **(B)** Female.

The annual transition probabilities from frailty to robust by childhood SES for men and women are depicted in Figure 2. It is apparent that as individuals age, the transition probabilities from frailty to robust decrease, regardless of gender. When examining male participants, those with low childhood SES experience a decrease from 20% at age 65 to 2.13% at age 95, while those with medium childhood SES decrease from 21% at age 65 to 2.27% at age 95, and those with high childhood SES decrease from 22% at age 65 to 2.37% at age 95. Notably, the transition probabilities for male participants tend to converge at age 95. Similarly, female participants exhibit a similar trend, with lower transition probabilities compared to their male counterparts in the same categories. It is evident that advantageous childhood SES has a protective effect on the recovery rate from frailty to robust, with the recovery rate for females being lower than that for males. However, with increasing age, the recovery rates tend to converge, regardless of gender.

**Figure 3:**
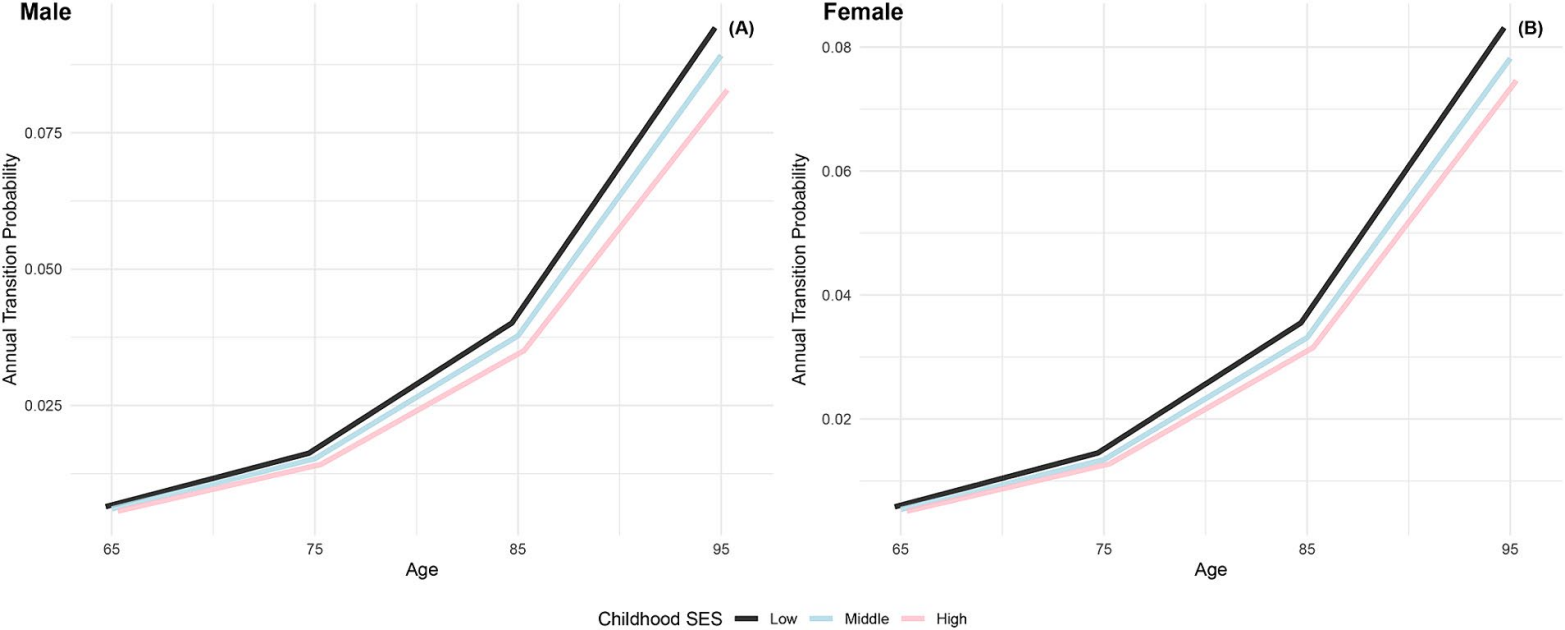
Annual transition probabilities from robust to mortality based on childhood SES, CLHLS 1998–2018. **(A)** Male. **(B)** Female.

Figure 3 illustrates the increasing annual transition probabilities from robust to dead by childhood SES for both genders as individuals age. Male participants with low, medium, and high childhood SES experience incremental increases in annual transition probabilities from robust to dead, with the lowest probability for the most favorable childhood SES category and the highest for the least favorable, mirroring the trend observed in female participants. Additionally, female participants across all age groups demonstrate lower annual transition probabilities from robust to dead than their male counterparts in the same categories, aligning with previous research conclusions that females have poorer health conditions but generally live longer than males (66, 76). Furthermore, the differences in transition probabilities from robust to dead among participants in the three childhood SES categories show an increasing trend as individuals age, providing preliminary evidence of the protective effect of advantageous childhood SES on the risk of death (29).

**Figure 4:**
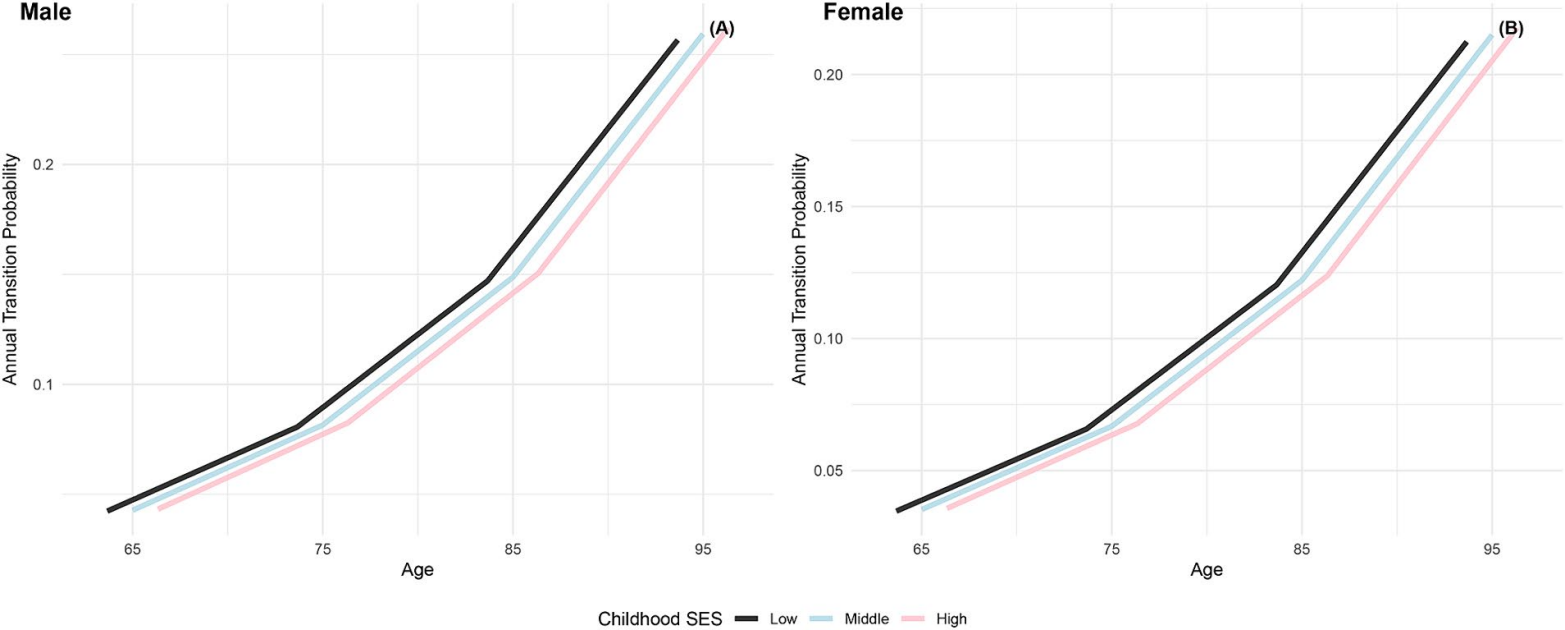
Annual transition probabilities from frailty to mortality based on childhood SES, CLHLS 1998–2018. **(A)** Male. **(B)** Female.

The annual transition probabilities from frailty to death by childhood SES for men and women are depicted in Figure 4. As age increases, the transition probabilities show an upward trend across all childhood SES categories. When considering gender, the annual transition probabilities from frailty to death for male participants in the three childhood SES categories (low, medium, high) decreased sequentially, while female participants exhibited a similar pattern. Notably, female participants in all age groups showed lower transition probabilities compared to their male counterparts in the same categories, indicating a potential longevity advantage for females (66, 76). Moreover, the differences in transition probabilities from frailty to death among participants in the three childhood SES categories show a slight widening trend with increasing age, suggesting a potential protective effect of favorable childhood SES on the risk of death (29).

#### 3.2.2 Annual transition probability by life course SES

**Figure 5:**
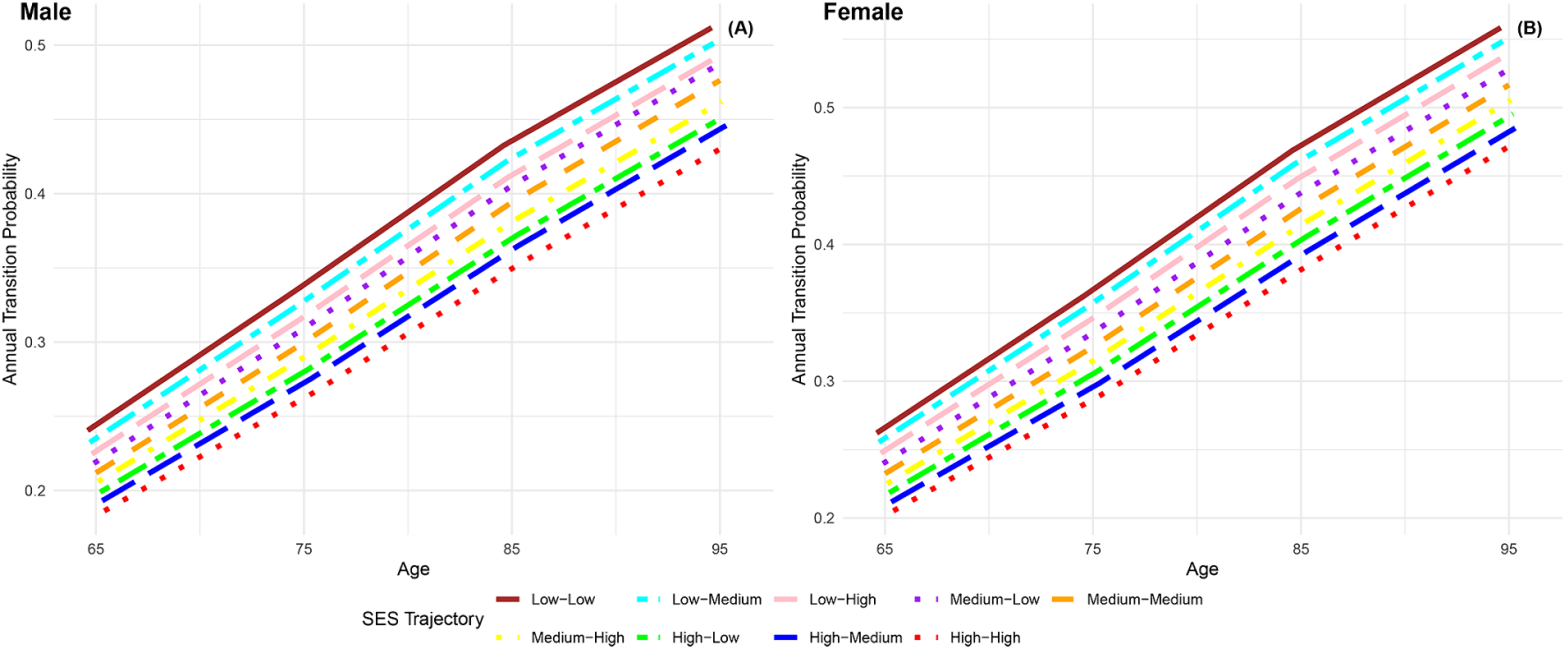
Annual transition probabilities from robust to frailty based on life-course SES trajectory, CLHLS 1998–2018. **(A)** Male. **(B)** Female.

The findings presented in Figure 5 depict the annual transition probabilities from robust to frailty based on life-course SES trajectory for both men and women. As participants age, there is a discernible increasing trend in transition probabilities from robust to frailty across the nine SES trajectory categories. Irrespective of gender, participants in the nine categories of life-course SES trajectory (low-low, low-medium, low-high, medium-low, medium-medium, medium-high, high-low, high-medium, high-high) demonstrate a sequential decrease in transition probabilities, with the most favorable category (high-high) exhibiting the lowest transition probability and the least favorable category (low-low) showing the highest. Notably, female participants consistently exhibit higher transition probabilities than their male counterparts in the same categories, indicating a higher frailty rate for females. The substantial differences in annual transition probabilities from robust to frailty between the most and least favorable SES trajectory categories underscore the close connection between lifelong SES disadvantage and frailty rates.

When comparing male participants with low-high and high-high life-course SES trajectory, the transition probability is 3.83% higher at age 65 and 5.83% higher at age 95 for those with low-high trajectory, indicating a significantly higher frailty rate for individuals with low childhood SES compared to high childhood SES, with the gap widening with age. Similarly, when comparing male participants with low-low and high-low life-course SES trajectory, the transition probability is 4.15% higher at age 65 and 5.98% higher at age 95 for those with low-low trajectory, indicating a protective effect of high childhood SES on frailty rates among individuals with low adulthood SES. Lastly, when comparing male participants with low-low and low-high life-course SES trajectories, the transition probability was found to be 1.58% higher at age 65 and 2.03% higher at age 95 for those with the low-low trajectory. This, combined with the results of comparing male participants with life-course SES trajectories of low-low and high-low, indicates a stronger protective effect of high childhood SES compared to high adulthood SES on frailty rates. This suggests that the transition probability from robust to frailty is more influenced by childhood SES rather than adulthood SES. Similar conclusions can be drawn from female participants. These findings further validate the critical period model (10) and the accumulation model (13, 20, 21).

**Figure 6:**
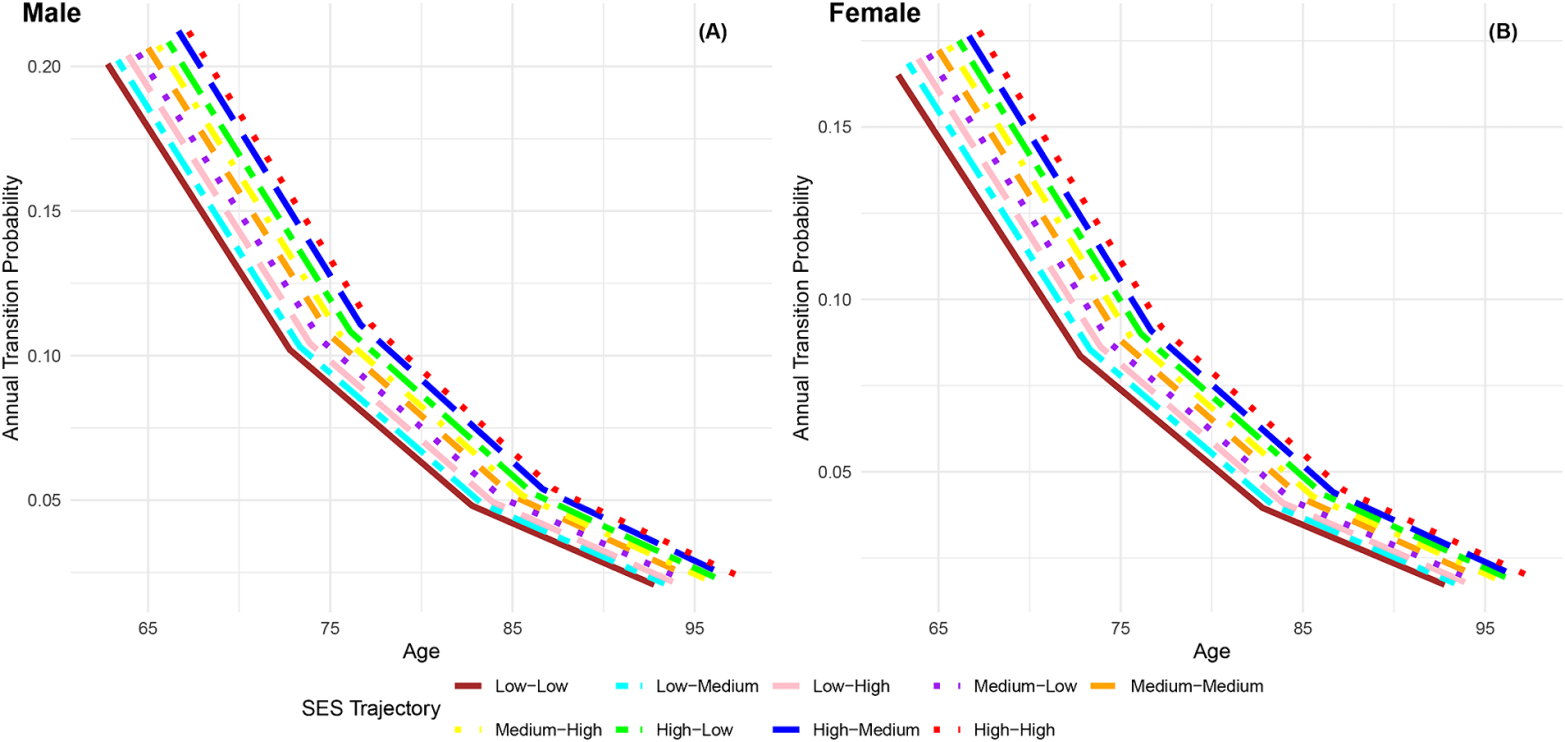
Annual transition probabilities from frailty to robust based on life-course SES trajectory, CLHLS 1998–2018. **(A)** Male. **(B)** Female.

The analysis depicted in Figure 6 demonstrates a consistent decline in annual transition probabilities from frailty to robust as individuals age across the nine life-course SES trajectory categories, regardless of gender. Irrespective of gender, the annual transition probabilities from frailty to robust increase sequentially across the nine life-course SES trajectory categories (low-low, low-medium, low-high, medium-low, medium-medium, medium-high, high-low, high-medium, high-high), with the most favorable category (high-high) exhibiting the highest transition probability, and the least favorable category (low-low) showing the lowest transition probability. Additionally, it is evident that across all age groups, the annual transition probabilities from frailty to robust for female participants in the nine life-course SES trajectory categories are lower than those for male participants in the same category, indicating a lower recovery rate for females compared to males. Regardless of gender, the difference in annual transition probabilities from frailty to robust between the most favorable (high-high) and the least favorable (low-low) categories of life-course SES trajectory underscores the protective effect of a favorable life-course SES trajectory on the recovery rate from frailty to robust. However, as age increases and physical function declines, the recovery rate diminishes, and the differences in recovery rates among the nine life-course SES trajectory categories tend to converge.

**Figure 7:**
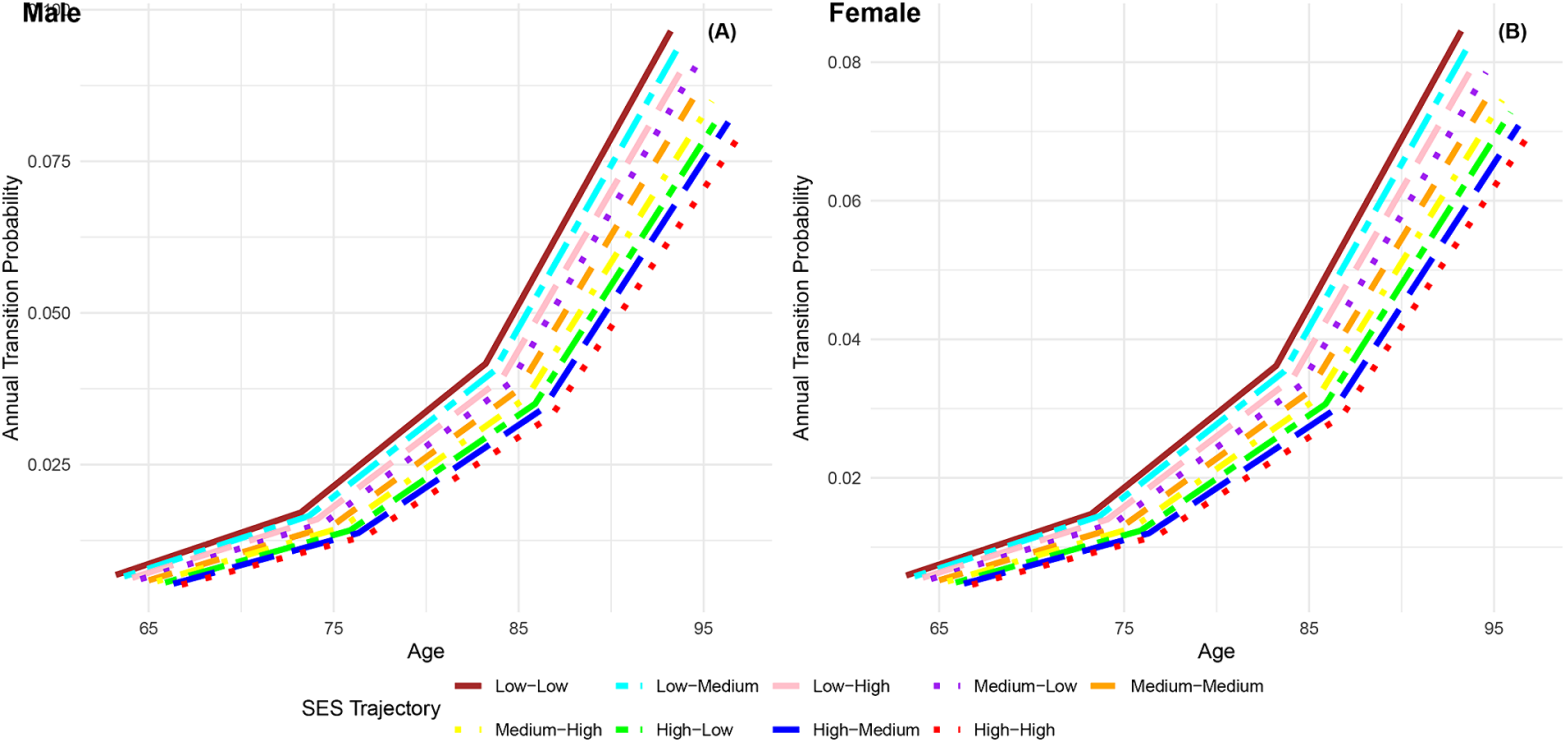
Annual transition probabilities from robust to mortality based on life-course SES trajectory, CLHLS 1998–2018. **(A)** Male. **(B)** Female.

Figure 7 presents the annual transition probabilities from robust to dead across different life-course SES trajectories for both men and women. The analysis reveals that, irrespective of gender, the annual transition probabilities from robust to dead increase with age for participants in the nine categories of life-course SES trajectory. Irrespective of gender, the annual transition probabilities from robust to dead for participants in the nine categories of life-course SES trajectory (low-low, low-medium, low-high, medium-low, medium-medium, medium-high, high-low, high-medium, high-high) decrease sequentially, with the most favorable category (high-high) exhibiting the lowest transition probability, and the least favorable category (low-low) showing the highest transition probability. Moreover, it is evident that across all age groups, the annual transition probabilities from robust to dead for female participants in the nine categories of life-course SES trajectory are lower than those for male participants in the corresponding categories, indicating a longer life expectancy for females (66, 76). Additionally, the analysis highlights that regardless of gender, the differences in transition probabilities from robust to dead among the nine categories of participants show an expanding trend with increasing age, emphasizing the protective effect of accumulated lifelong SES advantages on the risk of death (29). Furthermore, it is observed that a higher childhood SES provides stronger protection against the risk of death compared to a higher adult SES, suggesting that the transition probability from robust to dead is more influenced by childhood SES than adult SES.

**Figure 8:**
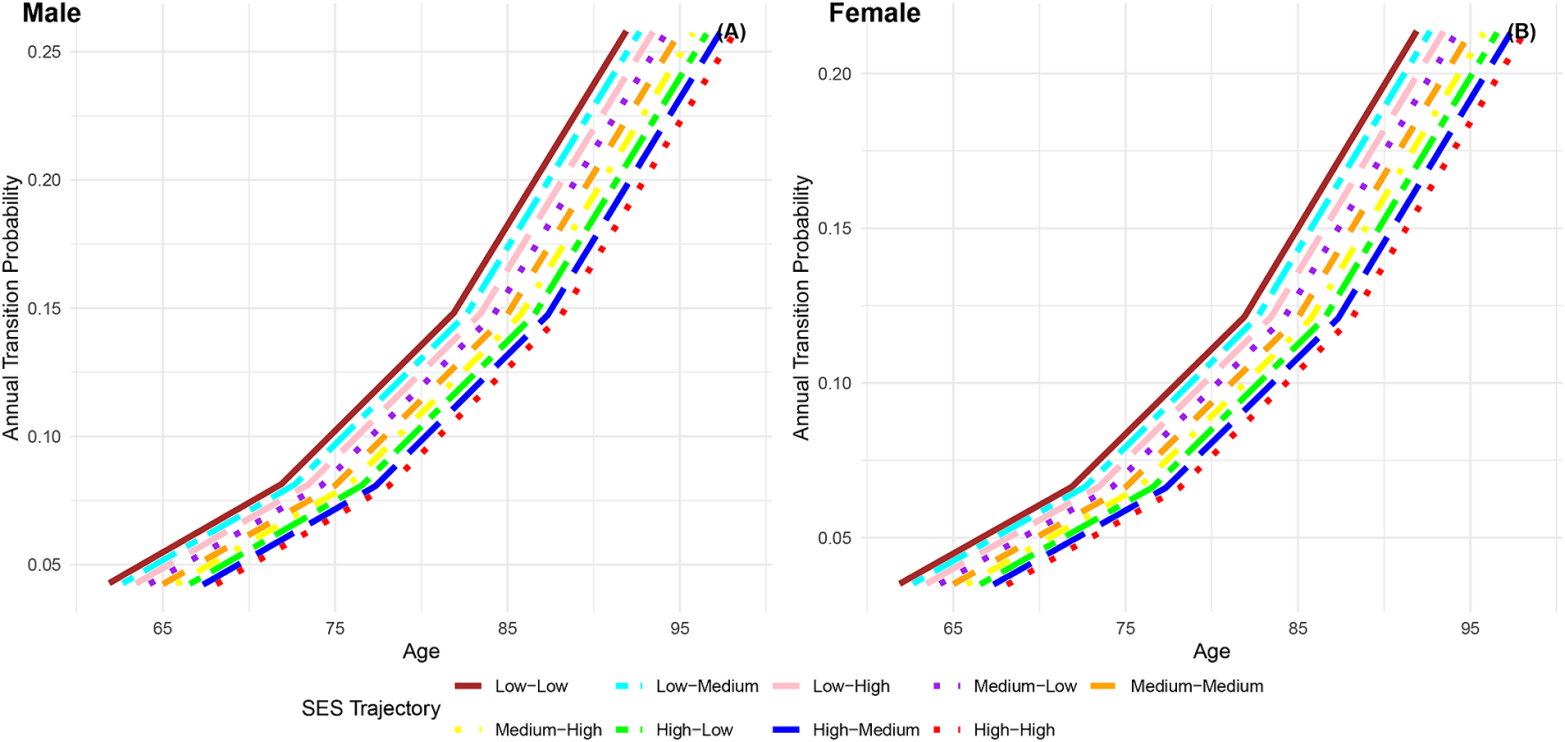
Annual transition probabilities from frailty to mortality based on life-course SES trajectory, CLHLS 1998–2018. **(A)** Male. **(B)** Female.

Figure 8 displays the annual transition probabilities from frailty to death by life-course SES trajectory for men and women. It is observed that regardless of gender, as age increases, the annual transition probabilities from frailty to death for participants in the nine categories of life-course SES trajectory show an increasing trend. Irrespective of gender, the annual transition probabilities from frailty to death for participants in the nine categories of life-course SES trajectory (low-low, low-medium, low-high, medium-low, medium-medium, medium-high, high-low, high-medium, high-high) decrease sequentially, with the most favorable category (high-high) exhibiting the lowest transition probability from frailty to death, and the least favorable category (low-low) showing the highest transition probability. Notably, at all age groups, the annual transition probabilities from frailty to death for female participants in the nine categories of life-course SES trajectory are lower than those for male participants in the corresponding categories, indicating that females generally live longer than males (66, 76). Additionally, the analysis highlights that regardless of gender, with increasing age, the differences in transition probabilities from frailty to death among the nine categories of participants show an expanding trend, emphasizing the protective effect of accumulated lifelong SES advantages on the risk of death (29).

### 3.3 Analysis of life expectancy

#### 3.3.1 Analysis of life expectancy by childhood SES

Table 2 presents a comparison of total, robust, and frailty life expectancy (LE) among Chinese men and women at ages 65 and 75, based on their childhood SES. Irrespective of gender, participants with low childhood SES exhibit the lowest total LE, followed by those with medium childhood SES, while the highest total LE is observed in participants with high childhood SES. At age 65, male participants in the three childhood SES categories demonstrate residual total LE of 14.34 years, 14.47 years, and 14.62 years, while female participants exhibit residual total LE of 15.50 years, 15.56 years, and 15.65 years. At age 75, male participants display residual total LE of 9.62 years, 9.74 years, and 9.91 years, while female participants show residual total LE of 10.55 years, 10.65 years, and 10.73 years. The robust LE exhibits a sequential increase, with the lowest observed in participants with low childhood SES, followed by those with medium childhood SES, and the highest in participants with high childhood SES. The robust LE proportion increases sequentially across the three childhood SES categories for both male and female participants. At age 65, the robust LE proportion for male participants in the low, medium, and high childhood SES categories is 33.05, 35.04, and 37.21% respectively, while for female participants, it is 26.77, 28.60, and 30.61%, respectively. At age 75, the estimated robust LE proportion for male participants in the three childhood SES categories is 31.50, 33.78, and 36.43% respectively, and for female participants, it is 25.50, 27.79, and 29.92%, respectively. Conversely, the frailty LE proportion demonstrates a sequential decrease, with the highest observed in participants with low childhood SES, followed by those with medium childhood SES, and the lowest in participants with high childhood SES.

**Table 2:** Childhood SES-stratified estimated robust, frailty, and total life expectancy (LE) at ages 65 and 75, including 95% confidence intervals (CLHLS 1998–2018).

Childhood SES	Total LE	Robust LE	Frailty LE	Proportion robust	Proportion frailty
Men					
Age 65					
Low childhood SES	14.34 [12.59, 14.52]	4.74 [4.10, 4.89]	9.59 [8.49, 9.74]	33.05%	66.95%
Medium childhood SES	14.47 [7.36, 14.67]	5.07 [2.29, 5.22]	9.40 [5.07, 9.56]	35.04%	64.96%
High childhood SES	14.62 [3.49, 14.93]	5.44 [0.71, 5.70]	9.19 [2.75, 9.38]	37.21%	62.79%
Age 75					
Low childhood SES	9.62 [9.11, 9.74]	3.03 [2.88, 3.13]	6.59 [6.22, 6.70]	31.50%	68.50%
Medium childhood SES	9.74 [7.33, 9.87]	3.29 [2.49, 3.39]	6.45 [4.83, 6.56]	33.78%	66.22%
High childhood SES	9.91 [5.17, 10.11]	3.61 [1.95, 3.79]	6.30 [3.22, 6.46]	36.43%	63.57%
Women					
Age 65					
Low childhood SES	15.50 [15.03, 15.73]	4.15 [3.96, 4.32]	11.35 [11.01, 11.56]	26.77%	73.23%
Medium childhood SES	15.56 [13.59, 15.79]	4.45 [3.74, 4.59]	11.11 [9.85, 11.32]	28.60%	71.40%
High childhood SES	15.65 [6.30, 15.93]	4.79 [1.55, 5.03]	10.86 [4.75, 11.11]	30.61%	69.39%
Age 75					
Low childhood SES	10.55 [10.33, 10.70]	2.69 [2.61, 2.79]	7.86 [7.66, 7.98]	25.50%	74.50%
Medium childhood SES	10.65 [10.26, 10.80]	2.96 [2.83, 3.05]	7.69 [7.40, 7.83]	27.79%	72.21%
High childhood SES	10.73 [9.44, 10.95]	3.21 [2.85, 3.37]	7.52 [6.56, 7.70]	29.92%	70.08%

① SES, socioeconomic status; ② LE, life expectancy; ③ Proportion robust, robust life expectancy accounted for total life expectancy; ④ Proportion frailty, frailty life expectancy accounted for total life expectancy.

Females exhibit a higher total LE than males, but their robust LE and robust LE proportion lag behind, reaffirming the notion that despite females generally outliving males, their health status tends to be poorer, marked by a higher frailty rate (66, 76). At age 65, male participants with low childhood SES have a robust LE proportion 4.15% lower than those with high childhood SES, and at age 75, this difference increases to 4.93%. Similarly, for females at age 65, the robust LE proportion for those with low childhood SES is 3.84% lower than those with high childhood SES, and at age 75, this difference grows to 4.42%. As individuals age, the disparity in robust LE proportion due to childhood SES disadvantage widens, once again affirming the critical period model (10).

#### 3.3.2 Analysis of life expectancy by life course SES

Table 3 presents the comparison of total, robust, and frailty LE among Chinese men and women at ages 65 and 75, across nine SES trajectories. Total LE increases sequentially across life-course SES trajectories, with the least favorable category (low-low) having the lowest total LE, and the most favorable category (high-high) having the highest. Male participants estimate residual total LE at 65 to be 14.097–14.575 years, and at 75 to be 9.49–10.13 years. Female participants estimate residual total LE at 65 to be 15.313–15.694 years, and at 75 to be 10.47–11.02 years. Irrespective of gender, participants’ robust LE in the nine life-course SES trajectory categories shows a sequential increase, with the least favorable category (low-low) exhibiting the lowest robust LE and the most favorable category (high-high) showing the highest robust LE.

**Table 3:** Estimated robust, frailty, and total life expectancy (LE) at ages 65 and 75 by life course SES trajectory, including 95% confidence intervals (CLHLS 1998–2018).

Life course SES trajectory (child–adult)	Total LE	Robust LE	Frailty LE	Proportion robust	Proportion frailty
**Men**					
Age 65					
Low–low	14.097 [12.913, 14.295]	4.465 [4.031, 4.634]	9.633 [8.857, 9.808]	31.67%	68.33%
Low–medium	14.155 [13.229, 14.361]	4.538 [4.212, 4.697]	9.617 [9.010, 9.773]	32.06%	67.94%
Low–high	14.216 [12.748, 14.392]	4.615 [4.050, 4.741]	9.601 [8.715, 9.754]	32.46%	67.54%
Medium–low	14.274 [10.616, 14.461]	4.671 [3.234, 4.815]	9.602 [7.382, 9.748]	32.72%	67.28%
Medium–medium	14.332 [7.053, 14.517]	4.747 [1.948, 4.897]	9.585 [5.091, 9.733]	33.12%	66.88%
Medium–high	14.368 [11.588, 14.584]	4.803 [3.723, 4.982]	9.565 [7.752, 9.739]	33.43%	66.57%
High–low	14.434 [7.206, 14.691]	4.892 [1.950, 5.094]	9.542 [5.179, 9.723]	33.89%	66.11%
High–medium	14.525 [2.886,14.799]	5.000 [0.155, 5.205]	9.524 [2.784, 9.772]	34.42%	65.58%
High–high	14.575 [4.125, 14.957]	5.080 [0.690, 5.396]	9.495 [3.437, 9.741]	34.85%	65.15%
Age 75					
Low–low	9.49 [8.92, 9.63]	2.91 [2.76, 3.01]	6.58 [6.14, 6.69]	30.66%	69.34%
Low–medium	9.57 [9.24, 9.69]	3.01 [2.89, 3.10]	6.56 [6.35, 6.66]	31.45%	68.55%
Low–high	9.65 [9.22, 9.77]	3.11 [2.97, 3.20]	6.53 [6.23, 6.63]	32.23%	67.77%
Medium–low	9.72 [8.17, 9.84]	3.20 [2.74, 3.28]	6.52 [5.43, 6.61]	32.92%	67.08%
Medium–medium	9.79 [6.59, 9.93]	3.30 [2.26, 3.39]	6.49 [4.33, 6.59]	33.71%	66.29%
Medium–high	9.87 [8.71, 10.02]	3.41 [3.01, 3.51]	6.46 [5.73, 6.59]	34.55%	65.45%
High–low	9.96 [7.99, 10.12]	3.52 [2.86, 3.65]	6.44 [5.17, 6.57]	35.34%	64.66%
High–medium	10.04 [1.09, 10.23]	3.63 [0.39, 3.75]	6.41 [0.70, 6.55]	36.16%	63.84%
High–high	10.13 [4.61, 10.34]	3.75 [1.72, 3.92]	6.38 [2.89, 6.55]	37.02%	62.98%
**Women**					
Age 65					
Low–low	15.313 [11.941, 15.521]	3.950 [3.003, 4.078]	11.363 [9.009, 11.559]	25.80%	74.20%
Low–medium	15.341 [14.486, 15.561]	4.013 [3.733, 4.149]	11.328 [10.695, 11.508]	26.16%	73.84%
Low–high	15.387 [14.718, 15.592]	4.072 [3.823, 4.226]	11.315 [10.847, 11.505]	26.46%	73.54%
Medium–low	15.434 [14.754, 15.680]	4.131 [3.912, 4.272]	11.303 [10.827, 11.505]	26.77%	73.23%
Medium–medium	15.485 [14.328, 15.729]	4.193 [3.795, 4.348]	11.292 [10.572, 11.530]	27.08%	72.92%
Medium–high	15.533 [12.917, 15.782]	4.257 [3.423, 4.452]	11.276 [9.470, 11.494]	27.41%	72.59%
High–low	15.579 [10.767, 15.850]	4.323 [2.724, 4.541]	11.256 [8.007, 11.489]	27.75%	72.25%
High–medium	15.635 [7.184, 15.969]	4.395 [1.427, 4.626]	11.241 [5.740, 11.513]	28.11%	71.89%
High–high	15.694 [6.085, 16.067]	4.471 [0.954,4.755]	11.223 [5.099, 11.514]	28.49%	71.51%
Age 75					
Low–low	10.47 [9.05, 10.61]	2.64 [2.31, 2.73]	7.83 [6.77, 7.94]	25.21%	74.79%
Low–medium	10.52 [10.21, 10.65]	2.72 [2.62, 2.81]	7.80 [7.58, 7.92]	25.86%	74.14%
Low–high	10.59 [10.38, 10.72]	2.82 [2.72, 2.91]	7.77 [7.61, 7.89]	26.63%	73.37%
Medium–low	10.66 [10.43, 10.80]	2.91 [2.79, 3.00]	7.75 [7.59, 7.88]	27.30%	72.70%
Medium–medium	10.73 [10.35, 10.88]	3.00 [2.85, 3.10]	7.72 [7.45, 7.85]	27.96%	72.04%
Medium–high	10.80 [10.43, 10.96]	3.10 [2.95, 3.22]	7.69 [7.41, 7.82]	28.70%	71.30%
High–low	10.86 [9.82, 11.02]	3.20 [2.88, 3.32]	7.66 [6.91, 7.82]	29.47%	70.53%
High–medium	10.94 [8.61, 11.11]	3.30 [2.72, 3.45]	7.64 [5.86, 7.79]	30.16%	69.84%
High–high	11.02 [4.79, 11.22]	3.41 [1.52, 3.57]	7.61 [3.27, 7.80]	30.94%	69.06%

① SES, socioeconomic status; ② LE, life expectancy; ③ Proportion Robust, robust life expectancy accounted for total life expectancy; ④ Proportion Frailty, frailty life expectancy accounted for total life expectancy.

The robust LE proportion among male and female participants in the nine categories of life-course SES trajectory (low-low, low-medium, low-high, medium-low, medium-medium, medium-high, high-low, high-medium, high-high) has exhibited a sequential increase. At the age of 65, the robust LE proportion for male participants in the nine categories of life-course SES trajectory was 31.67, 32.06, 32.46, 32.72, 33.12, 33.43, 33.89, 34.42, and 34.85%, respectively. Similarly, for female participants in the same categories, the robust LE proportion at the age of 65 was 25.80, 26.16, 26.46, 26.77, 27.08, 27.41, 27.75, 28.11, and 28.49%, respectively. Upon reaching the age of 75, the estimated robust LE proportion for male participants in the nine categories of life-course SES trajectory is 30.66, 31.45, 32.23, 32.92, 33.71, 34.55, 35.34, 36.16, and 37.02%, respectively. Correspondingly, for female participants in the same categories, the estimated robust LE proportion at the age of 75 is 25.21, 25.86, 26.63, 27.30, 27.96, 28.70, 29.47, 30.16, and 30.94%, respectively. In contrast, the frailty LE proportion of male and female participants in these categories decreases sequentially, with the least favorable category (low-low) having the highest frailty LE proportion and the most favorable category (high-high) having the lowest frailty LE proportion. The data reveals that across all nine categories, women exhibit a higher total LE compared to men, while their robust LE and robust LE proportion are lower than those of men. This reaffirms the notion that despite women generally outliving men, they experience poorer health conditions, with a heightened prevalence of frailty and illness (66, 76).

At the age of 65, the robust LE proportion of male participants following a low-low life-course socioeconomic status (SES) trajectory was found to be 3.18% lower than that of male participants with a high-high life-course SES trajectory. By the age of 75, the robust LE proportion of male participants with a low-low life-course SES trajectory had further decreased to 6.36% below that of their counterparts with a high-high life-course SES trajectory. Similarly, at the age of 65, the robust LE proportion of female participants with a low-low life-course SES trajectory was 2.69% lower than that of female participants with a high-high life-course SES trajectory, and by the age of 75, this proportion had decreased to 5.73% below that of female participants with a high-high life-course SES trajectory. These findings underscore the widening gap in robust LE proportion with age, emphasizing the cumulative impact of lifelong SES disadvantage, thus affirming the accumulation model (13, 20, 21).

At 65, male participants with a low-high life-course SES trajectory had a 2.39% lower robust LE proportion than those with a high-high trajectory. At 75, the difference increased to 4.79%. This suggests that men achieving high SES in adulthood, but with low childhood SES, had a significantly lower robust LE proportion, a difference that widened with age. Similar conclusions were drawn from female participants. For men with a low-low SES trajectory, the robust LE proportion was 2.22% lower at 65 and 4.68% lower at 75 compared to those with a high-low trajectory. This demonstrates the protective effect of high childhood SES on late-life robust LE proportion among men with low adult SES. Similar conclusions were drawn from female participants. At 65, male participants with a low-low SES trajectory had a 0.79% lower robust LE proportion than those with a low-high trajectory, increasing to 1.57% at 75. Comparing the robust LE proportion of male participants with a low-low life-course SES trajectory to that of male participants with a high-low life-course SES trajectory, this highlights the stronger protective effect of childhood SES on late-life robust LE proportion compared to adult SES. Similar conclusions were drawn from female participants, further validating the critical period model (10).

### 3.4 Enhancing result robustness through comparative analysis

#### 3.4.1 Validating the reliability of life expectancy calculations

There is a paucity of literature utilizing continuous time multi-state models to explore healthy longevity in later years from a life course perspective, with only two exceptions (4, 51). However, these two studies both rely on a single ADL to assess health status in later years, whereas this study employs Rockwood FI, comprising 38 indicators, to evaluate health status in later life. Consequently, the LE estimations from these two studies are not directly comparable to those of this study.

Given the scarcity of literature employing Rockwood FI and continuous time multi-state models to investigate healthy longevity in later years from a life course perspective, the sole exception applies Rockwood FI and continuous time multi-state models to study healthy longevity in later life, utilizing CLHLS data (61). Nonetheless, that study did not adopt a life course perspective. Nevertheless, as the indicators used to characterize health status in later years, models, and data sources in that study align with this study, the LE calculated in that study is compared with the results of this study to validate the reliability of the LE calculations presented here. That study computed LE by gender at age 65, resulting in a residual total LE of 14.16 years for males (Robust LE: 4.71 years, Frailty LE: 9.45 years) and 15.60 years for females (Robust LE: 3.58 years, Frailty LE: 12.02 years).

The above calculations are largely in line with those in this study. At age 65, the average residual total LE for male participants in the three childhood SES categories is 14.47 years, with Robust LE at 5.08 years and Frailty LE at 9.39 years. For female participants in the three childhood SES categories, the average residual total LE is 15.57 years, Robust LE is 4.46 years, and Frailty LE is 11.11 years. Similarly, at age 65, male participants in the nine life-course SES trajectory categories demonstrate an average residual total LE of 14.33 years, with Robust LE at 4.75 years and Frailty LE at 9.57 years. Female participants in the nine trajectory categories exhibit an average residual total LE of 15.49 years, Robust LE at 4.2 years, and Frailty LE at 11.29 years. This affirms the reliability of the LE calculations in this study.

#### 3.4.2 Enhancing result robustness through missing values removal

To enhance the response rate, this study employs multiple imputation by chained equations to impute missing data in childhood SES and adulthood SES indicators (4). Given the relatively limited number of missing values in each indicator, data points with missing values in childhood SES and adulthood SES are removed to assess result robustness. After the removal of missing values, the analytical sample comprises 32,432 participants, resulting in a response rate of 73.21%, with an average age of 88.18 years (SD: 11.14) and a male representation of 42%. The distribution of participants across low, medium, and high childhood SES categories is 66.89, 21.23, and 11.89%, respectively. Correspondingly, the distribution across low, medium, and high adulthood SES categories is 51.70, 33.26, and 15.04%, respectively. The distribution of participants among the nine categories of life-course SES trajectory (low-low, low-medium, low-high, medium-low, medium-medium, medium-high, high-low, high-medium, high-high) is 41.14, 20.03, 5.72, 8.91, 8.44, 3.87, 1.65, 4.79, and 5.45%, respectively. Notably, the age and gender distributions, along with the distributions in childhood SES, adulthood SES, and life course SES, exhibit consistency with the distributions before the deletion of missing values.

Following the removal of missing data, annual transition probabilities by childhood SES are illustrated in Supplementary Figures S3–S6, while annual transition probabilities by life course SES are delineated in Supplementary Figures S7–S10. The analysis of life expectancy by childhood SES is featured in Supplementary Table S4, and the analysis of life expectancy by life course SES is detailed in Supplementary Table S5. The study findings have demonstrated consistency with the outcomes prior to the deletion of missing values, affirming the robustness of the results. Given the potential widening of confidence intervals with a reduced estimation sample, precedence is given to utilizing the dataset without removed missing values as the principal outcomes.

## 4 Discussion

Studying the healthy longevity of older people in China from a life course perspective holds exceptional significance. This article, grounded in the life course approach, employs multi-state models to assess late-life health status using the Rockwood Frailty Index (FI), comprising 38 indicators. Leveraging the extensive CLHLS survey data spanning from 1998 to 2018, this research comprehensively examines how childhood SES and adult SES collectively shape late-life healthy longevity. By addressing the constraints of prior studies with limited sample sizes or brief durations (4), reliance on singular indicators for gauging late-life health status (4, 51), and models neglecting the interplay between disability incidence, recovery rate, and mortality rate (29, 30), this study makes a contribution to the field.

In China, older people demonstrate an upward social mobility trend from childhood to adulthood, with 29.7% experiencing upward mobility, while only 15.2% experience downward mobility, a proportion significantly lower than that of upwardly mobile participants. The multi-state transition probability forms the basis for estimating life expectancy. This study reveals that, irrespective of gender and the categorization of older people by childhood SES into three groups or by life-course SES trajectory into nine groups, the transition probabilities show an upward trend for robust–frailty, robust–dead, and frailty–dead with age, while the probability of frailty–robust exhibits a downward trend. Regardless of gender and the division of older people into three categories (low, medium, high) based on childhood SES or into nine categories (low-low, low-medium, low-high, medium-low, medium-medium, medium-high, high-low, high-medium, high-high) based on life-course SES trajectory, the transition probabilities of robust–frailty, robust–dead, and frailty–dead all decrease sequentially across different categories, while the probability of frailty–robust increases sequentially across different categories. Favorable childhood SES and the accumulation of lifelong SES advantages have a protective effect on frailty incidence, recovery rate, and mortality risk, with high childhood SES exerting a stronger influence than high adult SES.

Regardless of gender, whether older people are classified into three groups (low, medium, high) based on childhood SES or into nine groups (low-low, low-medium, low-high, medium-low, medium-medium, medium-high, high-low, high-medium, high-high) based on life-course SES trajectory, total LE, robust LE, and robust LE proportion exhibit sequential increases across different categories, while frailty LE shows sequential decreases across different categories. Favorable childhood SES and the accumulation of lifelong SES advantages exert a significant protective effect on late-life robust LE proportion, with high childhood SES demonstrating a stronger protective influence than high adult SES. The frailty incidence rate and total LE are higher in women than in men (40, 41), but the recovery rate, mortality risk, robust LE, and robust LE proportion are lower in women, indicating that despite women generally living longer than men, their health status is poorer (66, 76).

This study substantiates the critical period model (10) and accumulation model (13, 20, 21). Unlike prior research (4), our findings reveal that a higher childhood SES exerts a more potent protective influence on late-life healthy longevity than a higher adult SES. The policy implications of our study suggest that despite substantial enhancements in the living standards of most older Chinese people during adulthood and old age (4), these improvements do not counterbalance the adverse impact of childhood SES disadvantage on late-life healthy longevity (29). This underscores the crucial need for early intervention in late-life healthy longevity, as exposure to adverse conditions in early life is irreversible (12, 29, 43), and significantly contributes to late-life health inequality.

Our study’s findings align with various previous studies, highlighting the critical role of childhood SES in determining long-term health outcomes. Data from the Survey of Health, Aging, and Retirement in Europe (SHARE) indicated higher frailty levels among immigrants from LMICs in Nordic and Western Europe (36). Similarly, the 1958 British birth cohort linked lower childhood SES to higher FI scores (37), and a study on UK twins found an inverse relationship between father’s occupational class and participants’ FI (38). Research using SHARE and the China Health and Retirement Longitudinal Study (CHARLS) data showed that three or more childhood adversities increased FI levels (43), emphasizing the global nature of these effects. Our results support the critical period model (10), consistent with various studies. The Health and Retirement Study (HRS) in the U.S. linked childhood financial hardship to multimorbidity (12) and lower cognitive function (14). Research from the Utah Population Database found that higher childhood SES is associated with better morbidity trajectories and survival rates (15). These studies reinforce our conclusion that early-life socioeconomic conditions profoundly impact health trajectories. Additionally, our research indicates that high childhood SES has a greater impact than high adult SES. A British birth cohort study supports this, linking early-life SES to frailty, with adult SES only partially explaining this relationship (37). This underscores the need for early-life interventions to break the cycle of disadvantage.

The accumulation model validated in our study is also supported by several prior studies. For instance, a public health survey in Sweden linked lifelong economic stress to poor self-rated health (13). The HRS indicated that higher cumulative SES correlates with better cognitive function (14). A Life History survey in France associated lifelong socioeconomic disadvantage with increased morbidity ratios (22). Similarly, evidence from Sweden highlighted the impact of lifelong SES on health trajectories in older adult (26). These findings demonstrate that cumulative socioeconomic conditions throughout life significantly influence health outcomes, emphasizing the need for sustained socioeconomic support. Furthermore, while women tend to live longer than men, their overall health is worse. This aligns with findings from a cohort study in Germany (40), research in five major Latin American cities (41), and the HRS cohort in the U.S. (66). These studies, along with our findings, highlight the need for gender-specific health interventions to address unique health challenges faced by women. Despite extensive research from HICs, evidence from LMICs is limited, and these studies have not used continuous-time multi-state models. Thus, our study provides a valuable addition to the existing literature.

Given these findings, it is imperative for governmental authorities to strengthen nutritional and health initiatives aimed at disadvantaged children, improve the nutritional and health status of children in impoverished communities and households by executing early childhood nutrition enhancement initiatives (51), and further narrow the urban–rural gap. It is imperative to emphasize the implementation of comprehensive intervention strategies at multiple levels, including education, healthcare accessibility, and food provision, in order to develop effective strategies for promoting child health. Our research highlights the significance of approaching the topic of healthy aging from a life course perspective. We recommend that policymakers embrace a proactive strategy to enhance early-life nutrition and promote health among at-risk populations.

This study has several potential limitations. Firstly, due to the inability of the multi-state life table model to accommodate time-varying covariates, and our deliberate exclusion of variables susceptible to frailty, we did not incorporate indicators of current health behaviors or living arrangements as covariates. This decision was made because changes in health behaviors, such as smoking or physical activity, may be responses to changes in frailty (30), and these variables are also subject to change over time. Our study focuses on exploring the observable social differentiation in healthy longevity. We do not draw conclusions regarding the causal relationship between SES at various life stages and health outcomes in later life (4). Secondly, while the retrospective information was deemed reasonably reliable (64), the assessment of childhood SES relied on retrospective self-reports, inevitably introducing recall bias. However, the approach to mitigate recall bias involves enlarging the sample size, and the substantial sample size of the cohort data used in this study, 37,264 participants, can significantly mitigate the impact of recall bias on the research findings. Thirdly, individuals with extremely disadvantaged childhood SES may be absent from our study sample due to premature death. Although our study’s model incorporates age variable, the estimates derived from our sample may still be subject to survivor bias, potentially underestimating the influence of childhood SES on late-life healthy longevity (77).

## 5 Conclusion

Favorable childhood SES and lifelong accumulation of SES advantages protect against frailty morbidity, improve recovery rate, reduce mortality risk, and increase total LE, robust LE, and robust LE proportion. Moreover, high childhood SES exerts a stronger protective effect than high adult SES, signifying that enhancements in adult living conditions are inadequate to offset the detrimental impact of childhood SES disadvantage on late-life healthy longevity. This study substantiates the critical period model and accumulation model. Therefore, in order to promote healthy aging, it is imperative for policymakers to adopt a proactive strategy and implement targeted interventions addressing diverse health determinants in early life, encompassing aspects such as education, healthcare accessibility, and food supply.

The implications of this study extend to social, health, and public policy levels. Socially, addressing childhood SES disparities can lead to more equitable health outcomes across the lifespan. From a health perspective, early interventions can alleviate frailty and improve overall population health. At the public policy level, our findings advocate for policies that prioritize early-life health determinants, ensuring that resources are allocated to support disadvantaged populations from a young age, ultimately fostering a healthier, more equitable society.

However, this study has several potential limitations. Firstly, the multi-state life table model used does not accommodate time-varying covariates, and we excluded variables susceptible to frailty, such as current health behaviors and living arrangements. Secondly, the reliance on retrospective self-reports for childhood SES introduces recall bias, although the large sample size helps mitigate this issue. Lastly, the study may be subject to survivor bias, potentially underestimating the influence of childhood SES on late-life health outcomes.

Despite these limitations, this study offers valuable insights into how childhood SES and adult SES jointly shape late-life healthy longevity from a life course perspective, significantly contributing to a comprehensive understanding of the mechanisms that impact the healthy longevity of older people.

## Data Availability

The datasets presented in this study can be found in online repositories. The names of the repository/repositories and accession number(s) can be found in the article/Supplementary material.

## Supplementary material

SUPPLEMENTARY FIGURE S1 Flowchart depicting participant involvement in the CLHLS from 1998 to 2018.

SUPPLEMENTARY FIGURE S2 Multi-State Model Illustrating Transitions in Frailty States.

SUPPLEMENTARY FIGURE S3 Annual Transition Probabilities from Robust to Frailty Based on Childhood SES After Removing Missing Values, CLHLS 1998-2018. **(A)** Male. **(B)** Female.

SUPPLEMENTARY FIGURE S4 Annual Transition Probabilities from Frailty to Robust Based on Childhood SES After Removing Missing Values, CLHLS 1998-2018. **(A)** Male. **(B)** Female.

SUPPLEMENTARY FIGURE S5 Annual Transition Probabilities from Robust to Mortality Based on Childhood SES After Removing Missing Values, CLHLS 1998-2018. **(A)** Male. **(B)** Female.

SUPPLEMENTARY FIGURE S6 Annual Transition Probabilities from Frailty to Mortality Based on Childhood SES After Removing Missing Values, CLHLS 1998-2018. **(A)** Male. **(B)** Female.

SUPPLEMENTARY FIGURE S7 Annual Transition Probabilities from Robust to Frailty Based on Life-Course SES Trajectory After Removing Missing Values, CLHLS 1998-2018. **(A)** Male. **(B)** Female.

SUPPLEMENTARY FIGURE S8 Annual Transition Probabilities from Frailty to Robust Based on Life-Course SES Trajectory After Removing Missing Values, CLHLS 1998-2018. **(A)** Male. **(B)** Female.

SUPPLEMENTARY FIGURE S9 Annual Transition Probabilities from Robust to Mortality Based on Life-Course SES Trajectory After Removing Missing Values, CLHLS 1998-2018. **(A)** Male. **(B)** Female.

SUPPLEMENTARY FIGURE S10 Annual Transition Probabilities from Frailty to Mortality Based on Life-Course SES Trajectory After Removing Missing Values, CLHLS 1998-2018. **(A)** Male. **(B)** Female.

SUPPLEMENTARY TABLE S1 Inventory of Components comprising the Frailty Index.

SUPPLEMENTARY TABLE S2 Inventory of Components Comprising the Mini-Mental State Examination (MMSE).

SUPPLEMENTARY TABLE S3 Results of Hypothesis Tests.

SUPPLEMENTARY TABLE S4 Childhood SES-Stratified Estimated Robust, Frailty, and Total Life Expectancy (LE) at Ages 65 and 75, Including 95% Confidence Intervals (CLHLS 1998-2018) After Removing Missing Values.

SUPPLEMENTARY TABLE S5 Estimated Robust, Frailty, and Total Life Expectancy (LE) at Ages 65 and 75 by Life Course SES Trajectory, Including 95% Confidence Intervals (CLHLS 1998-2018) After Removing Missing Values.

## References

[1] National Bureau of Statistics of China. Communiqué of the seventh National Population Census (no. 5). (2021). Available at: https://www.stats.gov.cn/english/PressRelease/202105/t20210510_1817190.html (Accessed December 6, 2023).

[2] United Nations. World population prospects (2022). Available at: https://population.un.org/wpp/ (Accessed December 6, 2023)

[3] Yang L Wang Z. Early-life conditions and cognitive function in middle-and old-aged Chinese adults: a longitudinal study. Int J Environ Res Public Health. (2020) 17:3451. doi: 10.3390/ijerph17103451, PMID: 32429157 PMC7277849

[4] Payne CF Xu KQ. Life course socioeconomic status and healthy longevity in China. Demography. (2022) 59:629–52. doi: 10.1215/00703370-9830687, PMID: 35292811

[5] McEniry M McDermott J. Early-life conditions, rapid demographic changes, and older adult health in the developing world. Biodemography Soc Biol. (2015) 61:147–66. doi: 10.1080/19485565.2015.1047488, PMID: 26266970 PMC4559852

[6] Peele ME. Domains of childhood disadvantage and functional limitation trajectories among midlife men and women in China. J Aging Health. (2020) 32:501–12. doi: 10.1177/0898264319834813, PMID: 30845868 PMC8754423

[7] Heikkinen E. A life course approach: research orientations and future challenges. Eur Rev Aging Phys Act. (2011) 8:7–12. doi: 10.1007/s11556-010-0069-2

[8] Pudrovska T Anikputa B. Early-life socioeconomic status and mortality in later life: an integration of four life-course mechanisms. J Gerontol Ser B. (2014) 69:451–60. doi: 10.1093/geronb/gbt122, PMID: 24496607 PMC3983914

[9] Delgado-Angulo EK Bernabé E. Comparing lifecourse models of social class and adult oral health using the 1958 National Child Development Study. Community Dent Health. (2015) 32:20–5. doi: 10.1922/CDH_3412Angulo06 26263588

[10] Barker DJ. Maternal nutrition, fetal nutrition, and disease in later life. Nutr Burbank Los Angel Cty Calif. (1997) 13:807–13. doi: 10.1016/s0899-9007(97)00193-7 9290095

[11] Bernabé E Suominen AL Nordblad A Vehkalahti MM Hausen H Knuuttila M. Education level and oral health in Finnish adults: evidence from different lifecourse models. J Clin Periodontol. (2011) 38:25–32. doi: 10.1111/j.1600-051X.2010.01647.x, PMID: 21058971

[12] Tucker-Seeley RD Li Y Sorensen G Subramanian SV. Lifecourse socioeconomic circumstances and multimorbidity among older adults. BMC Public Health. (2011) 11:313. doi: 10.1186/1471-2458-11-313, PMID: 21569558 PMC3118239

[13] Lindström M Hansen K Rosvall M. Economic stress in childhood and adulthood, and self-rated health: a population based study concerning risk accumulation, critical period and social mobility. BMC Public Health. (2012) 12:761. doi: 10.1186/1471-2458-12-761, PMID: 22962948 PMC3491002

[14] Lyu J Burr JA. Socioeconomic status across the life course and cognitive function among older adults: an examination of the latency, pathways, and accumulation hypotheses. J Aging Health. (2016) 28:40–67. doi: 10.1177/0898264315585504, PMID: 26006338

[15] Zimmer Z Hanson HA Smith K. Childhood socioeconomic status, adult socioeconomic status, and old-age health trajectories: connecting early, middle, and late life. Demogr Res. (2016) 34:285–320. doi: 10.4054/DemRes.2016.34.10

[16] Hayward MD Gorman BK. The long arm of childhood: the influence of early-life social conditions on men’s mortality. Demography. (2004) 41:87–107. doi: 10.1353/dem.2004.0005 15074126

[17] Pakpahan E Hoffmann R Kröger H. The long arm of childhood circumstances on health in old age: evidence from SHARELIFE. Adv Life Course Res. (2017) 31:1–10. doi: 10.1016/j.alcr.2016.10.003

[18] Boylan JM Cundiff JM Jakubowski KP Pardini DA Matthews KA. Pathways linking childhood SES and adult health behaviors and psychological resources in black and white men. Ann Behav Med Publ Soc Behav Med. (2018) 52:1023–35. doi: 10.1093/abm/kay006, PMID: 29546291 PMC6230971

[19] Ben-Shlomo Y Kuh D. A life course approach to chronic disease epidemiology: conceptual models, empirical challenges and interdisciplinary perspectives. Int J Epidemiol. (2002) 31:285–93. doi: 10.1093/ije/31.2.285 PMID: 11980781

[20] O’Rand AM Hamil-Luker J. Processes of cumulative adversity: childhood disadvantage and increased risk of heart attack across the life course. J Gerontol B Psychol Sci Soc Sci. (2005) 60:117–24. doi: 10.1093/geronb/60.special_issue_2.s117, PMID: 16251582

[21] Willson AE Shuey KM Glen E. Cumulative advantage processes as mechanisms of inequality in life course health. Am J Sociol. (2007) 112:1886–924. doi: 10.1086/512712

[22] Melchior M Lert F Martin M Ville I. Socioeconomic position in childhood and in adulthood and functional limitations in midlife: data from a nationally-representative survey of French men and women. Soc Sci Med. (2006) 63:2813–24. doi: 10.1016/j.socscimed.2006.07.029, PMID: 16962219

[23] Lindström M Fridh M Rosvall M. Economic stress in childhood and adulthood, and poor psychological health: three life course hypotheses. Psychiatry Res. (2014) 215:386–93. doi: 10.1016/j.psychres.2013.11.018, PMID: 24332463

[24] Ramsay SE Papachristou E Watt RG Lennon LT Papacosta AO Whincup PH. Socioeconomic disadvantage across the life-course and oral health in older age: findings from a longitudinal study of older British men. J Public Health Oxf Engl. (2018) 40:e423–30. doi: 10.1093/pubmed/fdy068, PMID: 29684223 PMC6540288

[25] Lee H. A life course approach to total tooth loss: testing the sensitive period, accumulation, and social mobility models in the health and retirement study. Community Dent Oral Epidemiol. (2019) 47:333–9. doi: 10.1111/cdoe.12463, PMID: 31115080 PMC9218921

[26] Harber-Aschan L Calderón-Larrañaga A Darin-Mattson A Hu X Fratiglioni L Dekhtyar S. Beyond the social gradient: the role of lifelong socioeconomic status in older adults’ health trajectories. Aging. (2020) 12:24693–708. doi: 10.18632/aging.202342, PMID: 33349620 PMC7803509

[27] Lunyera J Stanifer JW Davenport CA Mohottige D Bhavsar NA Scialla JJ. Life course socioeconomic status, allostatic load, and kidney health in black Americans. Clin J Am Soc Nephrol CJASN. (2020) 15:341–8. doi: 10.2215/CJN.08430719, PMID: 32075808 PMC7057315

[28] Hallqvist J Lynch J Bartley M Lang T Blane D. Can we disentangle life course processes of accumulation, critical period and social mobility? An analysis of disadvantaged socio-economic positions and myocardial infarction in the Stockholm heart epidemiology program. Soc Sci Med. (2004) 58:1555–62. doi: 10.1016/S0277-9536(03)00344-7, PMID: 14759698

[29] Wen M Gu D. The effects of childhood, adult, and community socioeconomic conditions on health and mortality among older adults in China. Demography. (2011) 48:153–81. doi: 10.1007/s13524-010-0003-2, PMID: 21394657 PMC3690195

[30] Zimmer Z Martin LG Nagin DS Jones BL. Modeling disability trajectories and mortality of the oldest-old in China. Demography. (2012) 49:291–314. doi: 10.1007/s13524-011-0075-7, PMID: 22246796

[31] Zhong Y Wang J Nicholas S. Gender, childhood and adult socioeconomic inequalities in functional disability among Chinese older adults. Int J Equity Health. (2017) 16:165. doi: 10.1186/s12939-017-0662-3 28865465 PMC5581446

[32] Zhang X Jiang X Sha M Zhou Q Li W Guo Y. Life-course pathways from childhood socioeconomic status to type 2 diabetes in mid-late Chinese adulthood. Sci Rep. (2021) 11:13051. doi: 10.1038/s41598-021-91768-1, PMID: 34158532 PMC8219676

[33] Zhang X Dai S Jiang X Huang W Zhou Q Wang S. The pathways from disadvantaged socioeconomic status in childhood to edentulism in mid-to-late adulthood over the life-course. Int J Equity Health. (2023) 22:150. doi: 10.1186/s12939-023-01865-y, PMID: 37553562 PMC10408210

[34] Kojima G Liljas AEM Iliffe S. Frailty syndrome: implications and challenges for health care policy. Risk Manag Healthc Policy. (2019) 12:23–30. doi: 10.2147/RMHP.S168750, PMID: 30858741 PMC6385767

[35] Mitnitski A Song X Rockwood K. Trajectories of changes over twelve years in the health status of Canadians from late middle age. Exp Gerontol. (2012) 47:893–9. doi: 10.1016/j.exger.2012.06.015, PMID: 22790020

[36] Brothers TD Theou O Rockwood K. Frailty and migration in middle-aged and older Europeans. Arch Gerontol Geriatr. (2014) 58:63–8. doi: 10.1016/j.archger.2013.07.008, PMID: 23993266

[37] Rogers NT Blodgett JM Searle SD Cooper R Davis DHJ Pinto Pereira SM. Early-life socioeconomic position and the accumulation of health-related deficits by midlife in the 1958 British birth cohort study. Am J Epidemiol. (2021) 190:1550–60. doi: 10.1093/aje/kwab038, PMID: 33595066 PMC8327203

[38] Young ACM Glaser K Spector TD Steves CJ. The identification of hereditary and environmental determinants of frailty in a cohort of UK twins. Twin Res Hum Genet Off J Int Soc Twin Stud. (2016) 19:600–9. doi: 10.1017/thg.2016.72 27719687

[39] Maharani A Didikoglu A O’Neill TW Pendleton N Canal MM Payton A. Education mediating the associations between early life factors and frailty: a cross-sectional study of the UK biobank. BMJ Open. (2023) 13:e057511. doi: 10.1136/bmjopen-2021-057511, PMID: 36863735 PMC9990643

[40] Stephan A-J Strobl R Schwettmann L Meisinger C Ladwig K-H Linkohr B. The times we are born into and our lifestyle choices determine our health trajectories in older age - results from the KORA-age study. Prev Med. (2020) 133:106025. doi: 10.1016/j.ypmed.2020.106025, PMID: 32061683

[41] Alvarado BE Zunzunegui M-V Béland F Bamvita J-M. Life course social and health conditions linked to frailty in Latin American older men and women. J Gerontol A Biol Sci Med Sci. (2008) 63:1399–406. doi: 10.1093/gerona/63.12.1399, PMID: 19126855

[42] Cao X Ma C Zheng Z He L Hao M Chen X. Contribution of life course circumstances to the acceleration of phenotypic and functional aging: a retrospective study. EClinicalMedicine. (2022) 51:101548. doi: 10.1016/j.eclinm.2022.101548, PMID: 35844770 PMC9284373

[43] Wang Q. Social contexts and cross-national differences in association between adverse childhood experiences and frailty index. SSM - Popul Health. (2023) 22:101408. doi: 10.1016/j.ssmph.2023.101408, PMID: 37128358 PMC10148028

[44] Hargrove TW Brown TH. A life course approach to inequality: examining racial/ethnic differences in the relationship between early life socioeconomic conditions and adult health among men. Ethn Dis. (2015) 25:313–20. doi: 10.18865/ed.25.3.313, PMID: 26674267 PMC4671423

[45] Leopold L. Cumulative advantage in an egalitarian country? Socioeconomic health disparities over the life course in Sweden. J Health Soc Behav. (2016) 57:257–73. doi: 10.1177/0022146516645926 27284078

[46] Chen Y Zhao Y. Widening or convergence, the trajectories of health inequalities induced by childhood SES across the life course: evidence from China. SSM - Popul Health. (2023) 21:101324. doi: 10.1016/j.ssmph.2022.101324, PMID: 36632049 PMC9827053

[47] House JS Lepkowski JM Kinney AM Mero RP Kessler RC Herzog AR. The social stratification of aging and health. J Health Soc Behav. (1994) 35:213–34. PMID: 7983335

[48] House JS Lantz PM Herd P. Continuity and change in the social stratification of aging and health over the life course: evidence from a nationally representative longitudinal study from 1986 to 2001/2002 (Americans’ changing lives study). J Gerontol B Psychol Sci Soc Sci. (2005) 60 Spec No) 60 Spec No 2:15–26. doi: 10.1093/geronb/60.special_issue_2.s15, PMID: 16251586

[49] McEniry M. Early-life conditions and older adult health in low- and middle-income countries: a review. J Dev Orig Health Dis. (2013) 4:10–29. doi: 10.1017/S2040174412000499, PMID: 23316272 PMC3540412

[50] Cai L Schenker N Lubitz J. Analysis of functional status transitions by using a semi-Markov process model in the presence of left-censored spells. J R Stat Soc Ser C Appl Stat. (2006) 55:477–91. doi: 10.1111/j.1467-9876.2006.00548.x

[51] Dong H Du C Wu B Wu Q. Multi-state analysis of the impact of childhood starvation on the healthy life expectancy of the elderly in China. Front Public Health. (2021) 9:690645. doi: 10.3389/fpubh.2021.690645, PMID: 34291030 PMC8287127

[52] Yi Z. Introduction to the Chinese longitudinal healthy longevity survey (CLHLS) In: Yi Z Poston DL Vlosky DA Gu D, editors. Healthy longevity in China: Demographic, socioeconomic, and psychological dimensions. Dordrecht: springer Netherlands (2008). 23–38.

[53] Gu D. General data quality assessment of the CLHLS In: Yi Z Poston DL Vlosky DA Gu D, editors. Healthy longevity in China: Demographic, socioeconomic, and psychological dimensions. Dordrecht: springer Netherlands (2008). 39–60.

[54] Dupre ME Gu D Warner DF Yi Z. Frailty and type of death among older adults in China: prospective cohort study. BMJ. (2009) 338:b1175. doi: 10.1136/bmj.b1175, PMID: 19359289 PMC2667569

[55] Zeng Y Feng Q Gu D Vaupel JW. Demographics, phenotypic health characteristics and genetic analysis of centenarians in China. Mech Ageing Dev. (2017) 165:86–97. doi: 10.1016/j.mad.2016.12.010, PMID: 28040447 PMC5489367

[56] Gu D Dupre ME Sautter J Zhu H Liu Y Yi Z. Frailty and mortality among Chinese at advanced ages. J Gerontol B Psychol Sci Soc Sci. (2009) 64:279–89. doi: 10.1093/geronb/gbn009, PMID: 19196691 PMC2655172

[57] Gu Y Wu W Bai J Chen X Chen X Yu L. Association between the number of teeth and frailty among Chinese older adults: a nationwide cross-sectional study. BMJ Open. (2019) 9:e029929. doi: 10.1136/bmjopen-2019-029929, PMID: 31640996 PMC6830605

[58] Searle SD Mitnitski A Gahbauer EA Gill TM Rockwood K. A standard procedure for creating a frailty index. BMC Geriatr. (2008) 8:24. doi: 10.1186/1471-2318-8-24, PMID: 18826625 PMC2573877

[59] Bennett S Song X Mitnitski A Rockwood K. A limit to frailty in very old, community-dwelling people: a secondary analysis of the Chinese longitudinal health and longevity study. Age Ageing. (2013) 42:372–7. doi: 10.1093/ageing/afs180 23232936

[60] Li S Fan W Zhu B Ma C Tan X Gu Y. Effects of age, period, and cohort on the prevalence of frailty in Chinese older adults from 2002 to 2014. Front Public Health. (2022) 10:935163. doi: 10.3389/fpubh.2022.935163, PMID: 36033734 PMC9412743

[61] Gao J Wang Y Xu J Jiang J Yang S Xiao Q. Life expectancy among older adults with or without frailty in China: multistate modelling of a national longitudinal cohort study. BMC Med. (2023) 21:101. doi: 10.1186/s12916-023-02825-7, PMID: 36927351 PMC10021933

[62] Shi G-P Ma T Zhu Y-S Wang Z-D Chu X-F Wang Y. Frailty phenotype, frailty index and risk of mortality in Chinese elderly population- Rugao longevity and ageing study. Arch Gerontol Geriatr. (2019) 80:115–9. doi: 10.1016/j.archger.2018.11.001, PMID: 30448694

[63] Gu D Feng Q Chen H Zeng Y. Chinese longitudinal healthy longevity survey (CLHLS) In: Gu D Dupre ME, editors. Encyclopedia of gerontology and population aging. Cham: Springer International Publishing (2021). 957–70.

[64] Luo Y Waite LJ. The impact of childhood and adult SES on physical, mental, and cognitive well-being in later life. J Gerontol B Psychol Sci Soc Sci. (2005) 60:S93–S101. doi: 10.1093/geronb/60.2.s93, PMID: 15746030 PMC2505177

[65] Zhang Z Liu J Li L Xu H. The long arm of childhood in China: early-life conditions and cognitive function among middle-aged and older adults. J Aging Health. (2018) 30:1319–44. doi: 10.1177/0898264317715975, PMID: 28645234

[66] Morton PM Ferraro KF. Early social origins of biological risks for men and women in later life. J Health Soc Behav. (2020) 61:503–22. doi: 10.1177/0022146520966364, PMID: 33205672 PMC7857845

[67] Luo Y Wen M. Can we afford better health? A study of the health differentials in China. Health (N Y). (2002) 6:471–500. doi: 10.1177/136345930200600404

[68] Zimmer Z Kwong J. Socioeconomic status and health among older adults in rural and urban China. J Aging Health. (2004) 16:44–70. doi: 10.1177/0898264303260440, PMID: 14979310

[69] Zhu H Xie Y. Socioeconomic differentials in mortality among the oldest old in China. Res Aging. (2007) 29:125–43. doi: 10.1177/0164027506296758

[70] Huang X Deng J Liu W. Sex differences in cognitive function among Chinese older adults using data from the Chinese longitudinal healthy longevity survey: a cross-sectional study. Front Public Health. (2023) 11:1182268. doi: 10.3389/fpubh.2023.1182268, PMID: 37457255 PMC10343959

[71] Pedersen J Bjorner JB. Worklife expectancy in a cohort of Danish employees aged 55-65 years - comparing a multi-state cox proportional hazard approach with conventional multi-state life tables. BMC Public Health. (2017) 17:879. doi: 10.1186/s12889-017-4890-7, PMID: 29141598 PMC5688741

[72] Zaninotto P Head J Steptoe A. Behavioural risk factors and healthy life expectancy: evidence from two longitudinal studies of ageing in England and the US. Sci Rep. (2020) 10:6955. doi: 10.1038/s41598-020-63843-6 32332825 PMC7181761

[73] Jackson C. Multi-state models for panel data: the msm package for R. J Stat Softw. (2011) 38:1–28. doi: 10.18637/jss.v038.i08

[74] van den Hout A Sum Chan M Matthews F. Estimation of life expectancies using continuous-time multi-state models. Comput Methods Prog Biomed. (2019) 178:11–8. doi: 10.1016/j.cmpb.2019.06.004, PMID: 31416539

[75] Turrell G Lynch JW Kaplan GA Everson SA Helkala E-L Kauhanen J. Socioeconomic position across the lifecourse and cognitive function in late middle age. J Gerontol B Psychol Sci Soc Sci. (2002) 57:S43–51. doi: 10.1093/geronb/57.1.s43 11773232

[76] Read JG Gorman BK. Gender and health inequality. Annu Rev Sociol. (2010) 36:371–86. doi: 10.1146/annurev.soc.012809.102535

[77] Tian F Meng SS Qiu P. Childhood adversities and mid-late depressive symptoms over the life course: evidence from the China health and retirement longitudinal study. J Affect Disord. (2019) 245:668–78. doi: 10.1016/j.jad.2018.11.028 30445392

