# Supplementary material for: Childhood and adult socioeconomic status influence on late-life healthy longevity: evidence from the Chinese longitudinal healthy longevity survey
Source: Front Public Health. 2024 Sep 30;12:1352937. doi: 10.3389/fpubh.2024.1352937 (PMC11471603; doi:10.3389/fpubh.2024.1352937)
Supplement: SUPPLEMENTARY FIGURE S1 — Flowchart depicting participant involvement in the CLHLS from 1998 to 2018. [file Data_Sheet_1.docx]

Supplementary Material

# Supplementary Figures


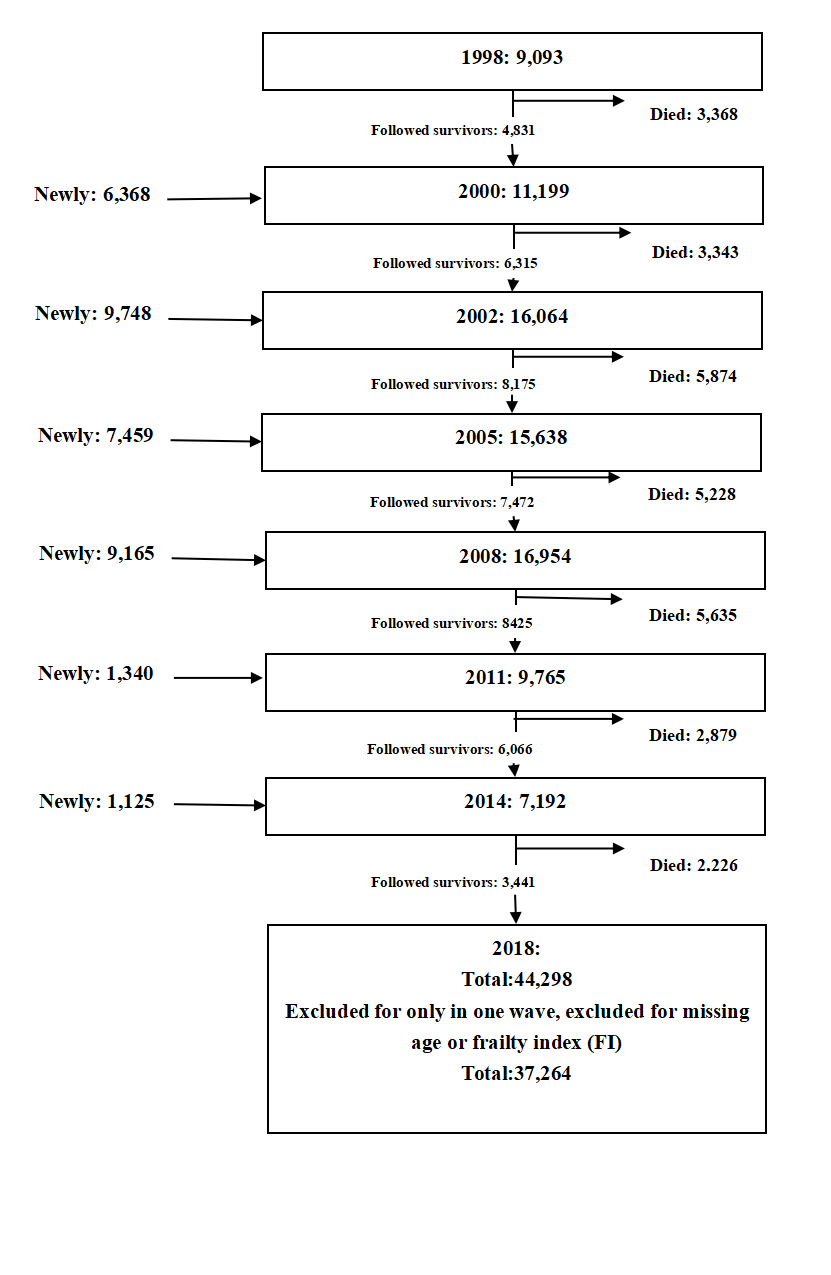


**Supplementary Figure 1.** Flowchart depicting participant involvement in the CLHLS from 1998 to 2018

**
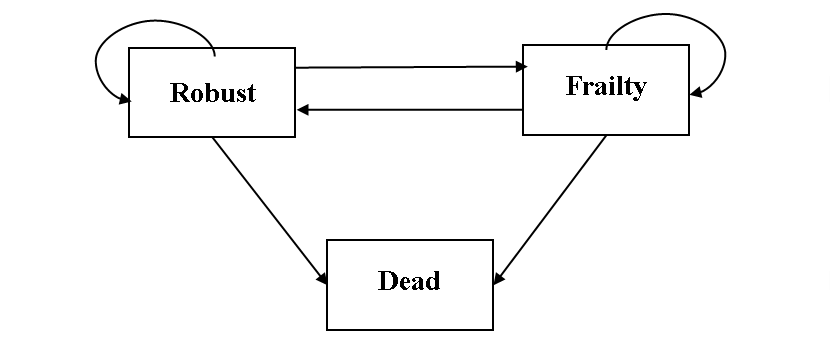
**

**Supplementary Figure 2.** Multi-State Model Illustrating Transitions in Frailty States

**
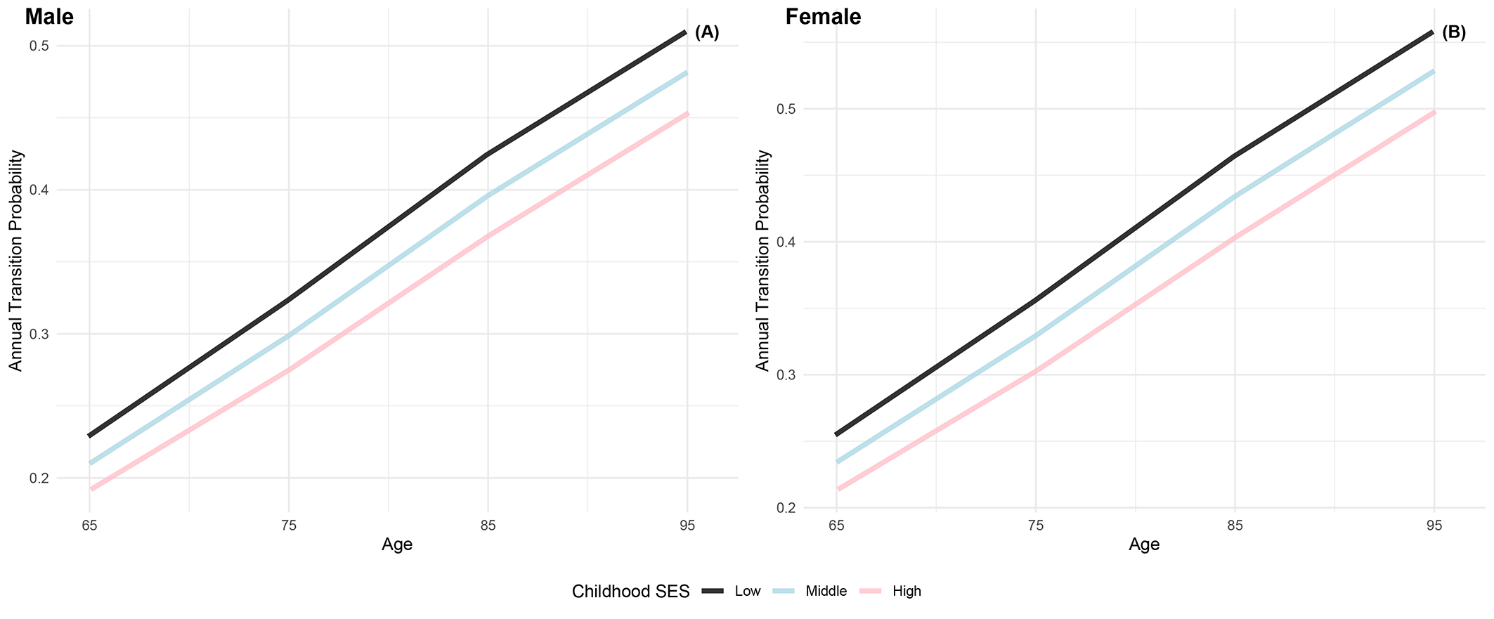
**

**Supplementary Figure 3.** Annual Transition Probabilities from Robust to Frailty Based on Childhood SES After Removing Missing Values, CLHLS 1998-2018. (A) Male. (B) Female.

**
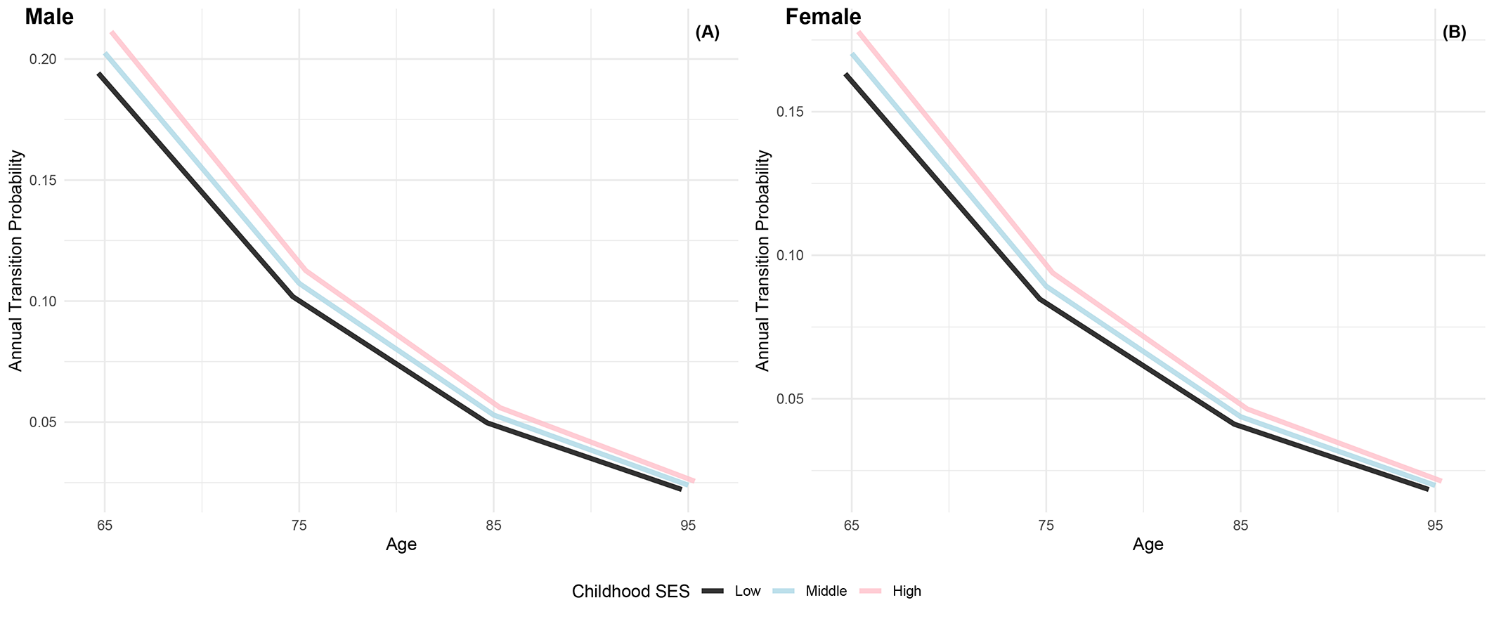
**

**Supplementary Figure 4.** Annual Transition Probabilities from Frailty to Robust Based on Childhood SES After Removing Missing Values, CLHLS 1998-2018. (A) Male. (B) Female.

**
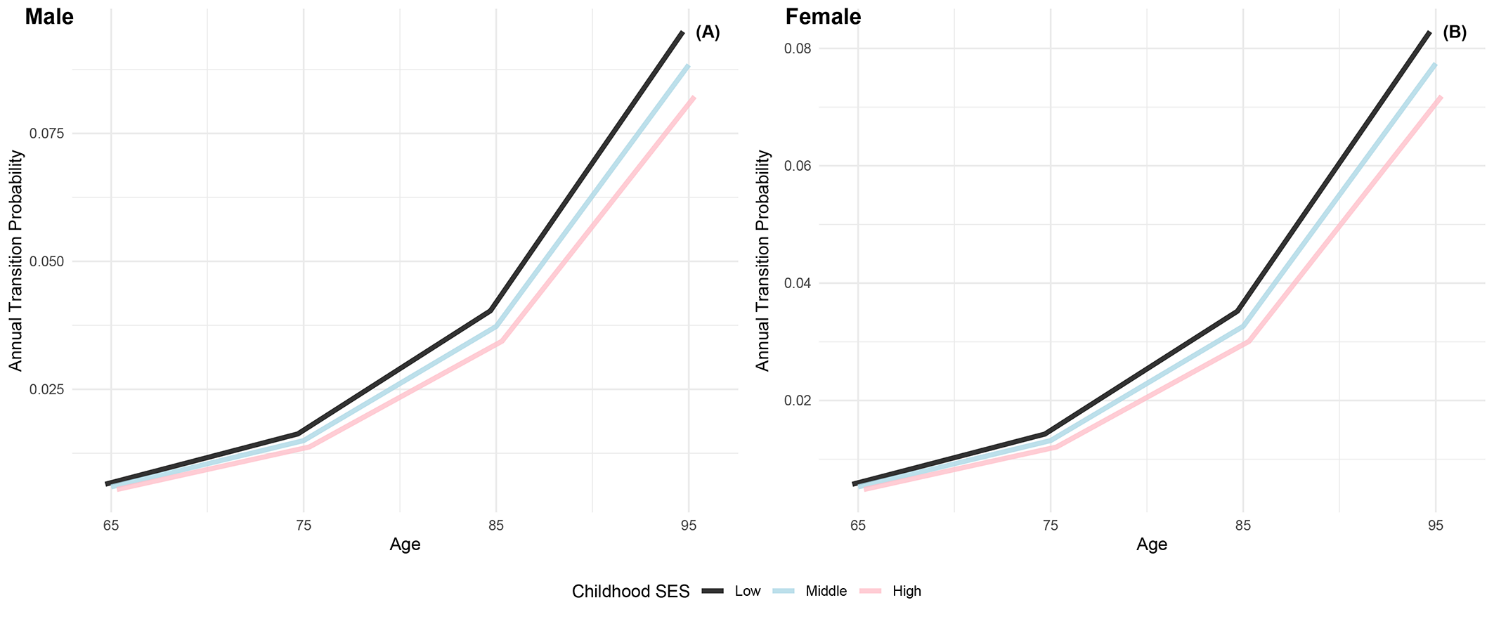
**

**Supplementary Figure 5.** Annual Transition Probabilities from Robust to Mortality Based on Childhood SES After Removing Missing Values, CLHLS 1998-2018. (A) Male. (B) Female.


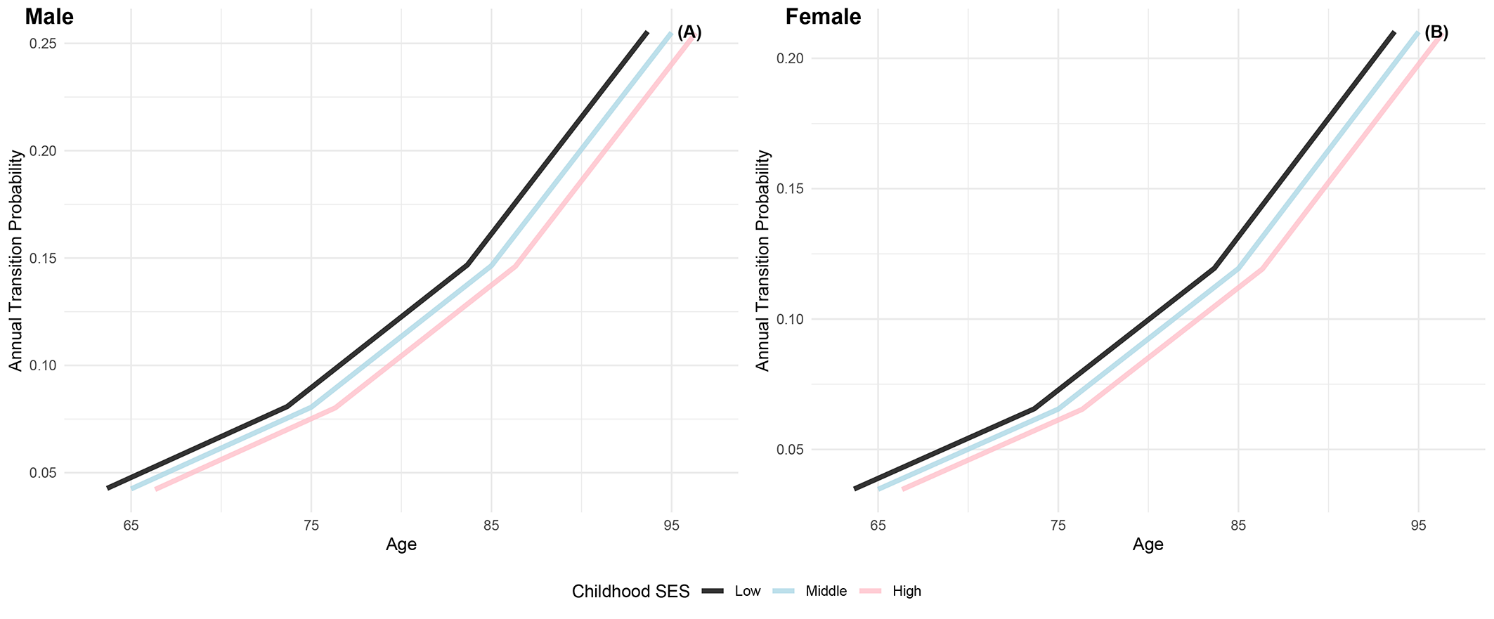


**Supplementary Figure 6.** Annual Transition Probabilities from Frailty to Mortality Based on Childhood SES After Removing Missing Values, CLHLS 1998-2018. (A) Male. (B) Female.


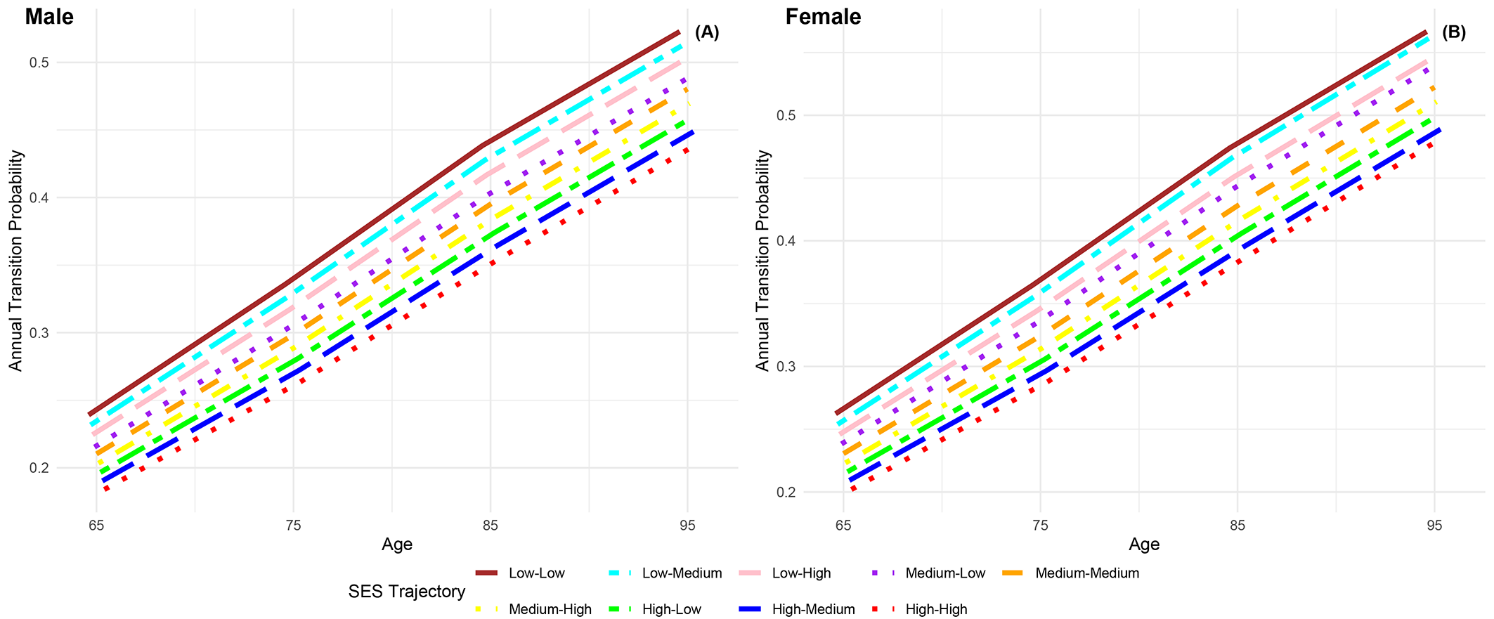


**Supplementary Figure 7.** Annual Transition Probabilities from Robust to Frailty Based on Life-Course SES Trajectory After Removing Missing Values, CLHLS 1998-2018. (A) Male. (B) Female.

**
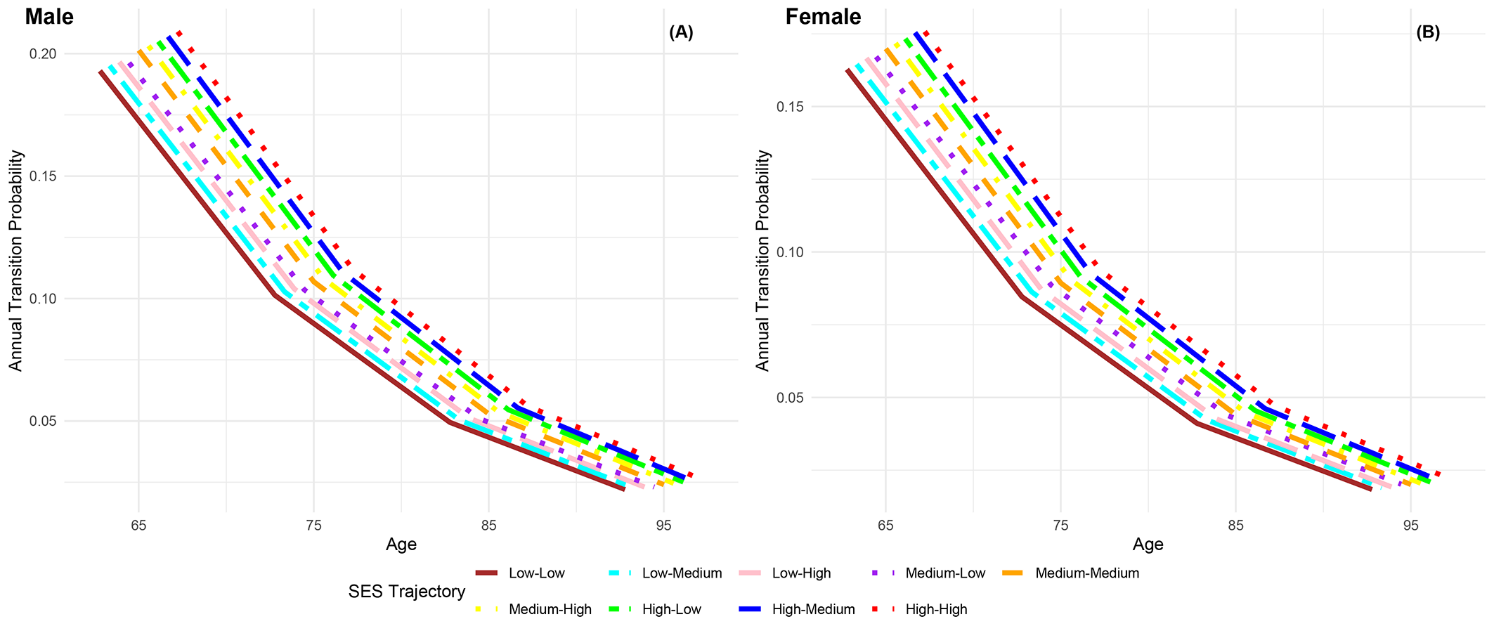
**

**Supplementary Figure 8.** Annual Transition Probabilities from Frailty to Robust Based on Life-Course SES Trajectory After Removing Missing Values, CLHLS 1998-2018. (A) Male. (B) Female.


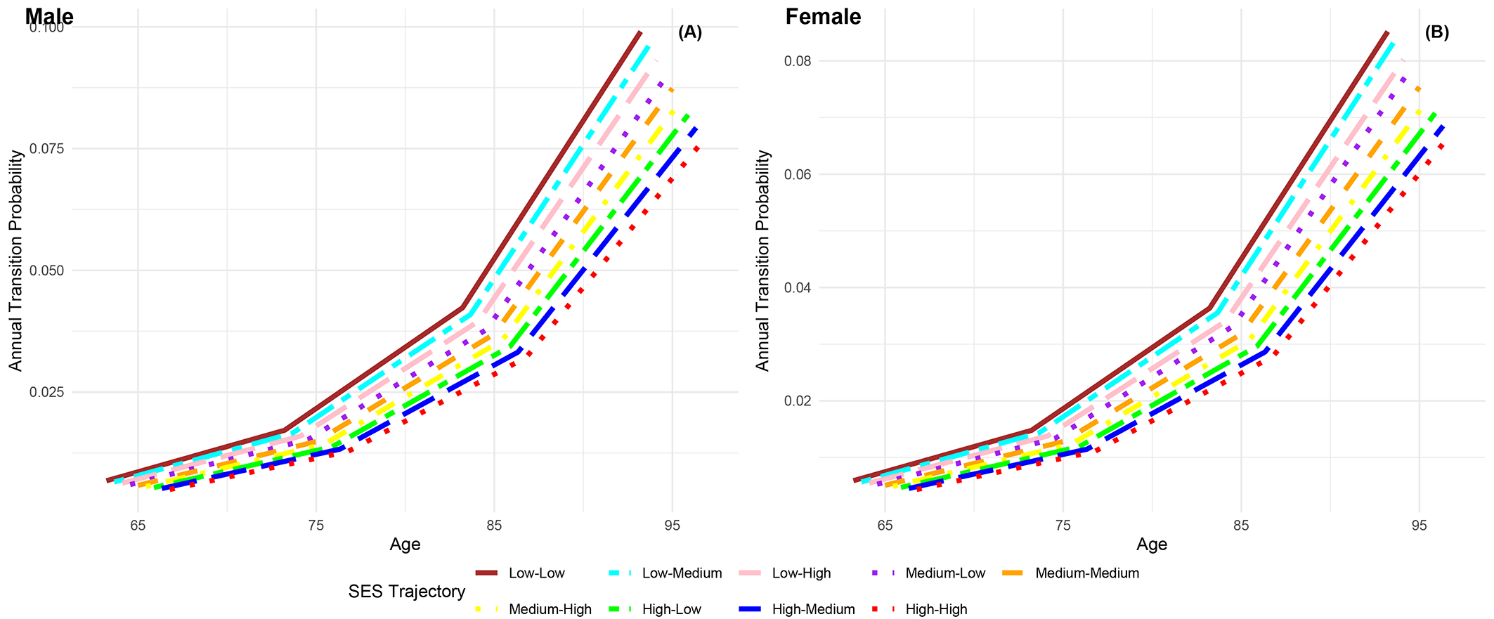


**Supplementary Figure 9.** Annual Transition Probabilities from Robust to Mortality Based on Life-Course SES Trajectory After Removing Missing Values, CLHLS 1998-2018. (A) Male. (B) Female.

**
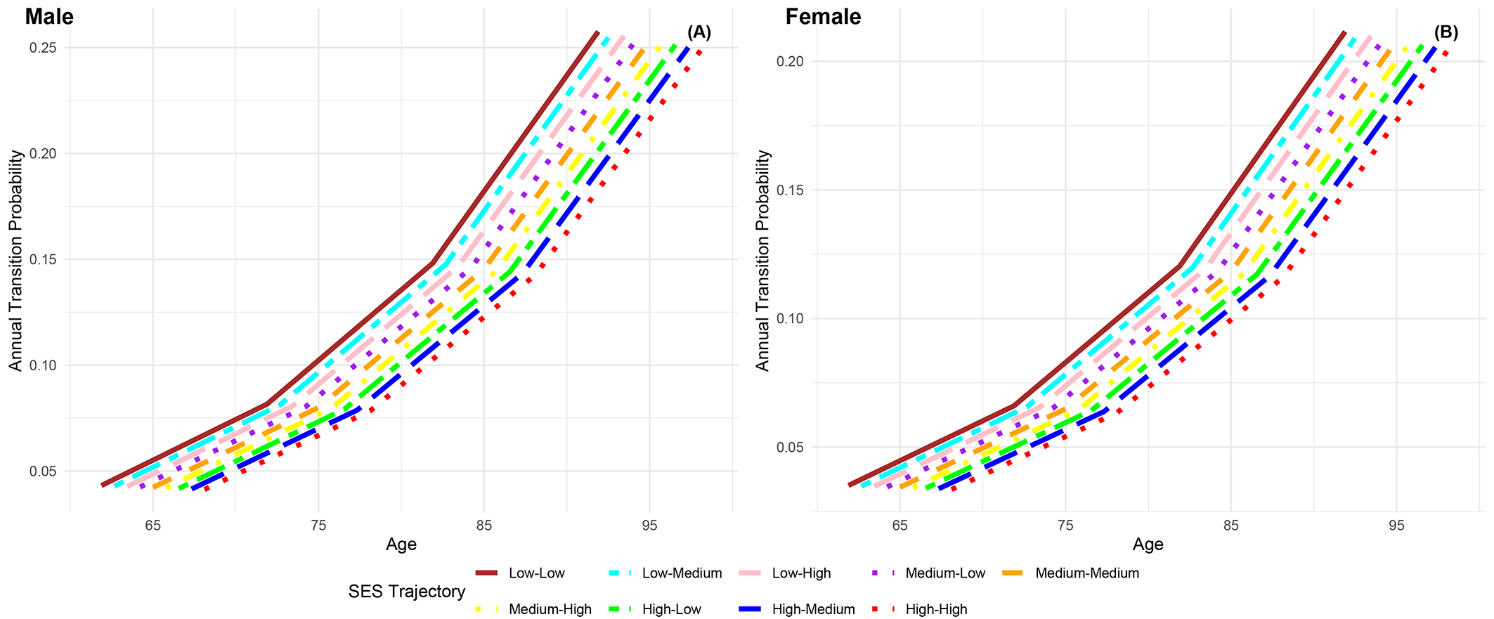
**

**Supplementary Figure 10.** Annual Transition Probabilities from Frailty to Mortality Based on Life-Course SES Trajectory After Removing Missing Values, CLHLS 1998-2018. (A) Male. (B) Female.

# Supplementary Tables

**Supplementary Table 1.** Inventory of Components comprising the Frailty Index

|  | Variables | Question No. | Values |
| --- | --- | --- | --- |
| 1 | Bathing | e1 | without assistance=0; assistance with 1 body part=0.5; assistance with >1 body part=1 |
| 2 | Dressing | e2 | without assistance=0; some assistance=0.5; unable to dress without assistance=1 |
| 3 | Toileting | e3 | without assistance=0; some assistance=0.5; unable to use toilet without assistance=1 |
| 4 | Indoor activities | e4 | without assistance=0; some assistance=0.5; a lot of assistance=1 |
| 5 | Continence | e5 | able=0; occasional incontinence=0.5; frequent incontinence=1 |
| 6 | Feeding | e6 | without assistance=0; some assistance=0.5; a lot of assistance=1 |
| 7 | Hand behind neck | g101 | right hand=0.5; left hand=0.5; both hands=0; neither hand=1 |
| 8 | Hand behind lower back | g102 | right hand=0.5; left hand=0.5; both hands=0; neither hand=1 |
| 9 | Able to stand up from sitting | g11 | yes, without using hands=0; yes, using hands=0.5; no=1 |
| 10 | Able to pick up a book from the floor | g13 | yes, standing=0; yes, sitting=0.5; no=1 |
| 11 | Able to use chopsticks to eat | g3 | yes=0; no=1 |
| 12 | Number of steps used to turn around a 360 degree turn without help | g14 | ≤4=0; >4=0.5; cannot turn around=1 |
| 13 | Visual function | g1 | can see and distinguish the break in the circle=0; can see but not distinguish the break in the circle=0.33; cannot see=0.67; blind=1 |
| 14 | Hearing ability | h1a | can hear without a hearing aid=0; can hear with a hearing aid=0.33; partly deaf, despite using a hearing aid=0.67; deaf=1 |
| 15 | Mini-Mental State Examination (MMSE) |  | 0:9=1; 10:17=0.67; 18:23=0.33; 24:30=0 |
| 16 | Self-reported health | b12 | very good=0; good=0.25; average=0.5; bad=0.75; very bad=1 |
| 17 | Feel fearful or anxious | b23 | always=1; often=0.75; sometimes=0.5; seldom=0.25; never=0 |
| 18 | Feel useless because of age | b26 | always=1; often=0.75; sometimes=0.5; seldom=0.25; never=0 |
| 19 | Look on the bright side of things | b21 | always=0; often=0.25; sometimes=0.5; seldom=0.75; never=1 |
| 20 | Keep my belongings neat and clean | b22 | always=0; often=0.25; sometimes=0.5; seldom=0.75; never=1 |
| 21 | Make own decisions | b25 | always=0; often=0.25; sometimes=0.5; seldom=0.75; never=1 |
| 22 | Cardiac rhythm | g6 | regular=0; irregular=1 |
| 23 | Housework at present | d10a | almost every day=0; not daily, but at least once a week=0.5; not weekly, but at least once a month=0.5; not monthly, but sometimes=0.5; never=1 |
| 24 | Interviewer rated-­health | h3 | surprisingly healthy=0; relatively healthy=0.33; moderately unhealthy=0.67; very unhealthy=1 |
| 25 | Number of times suffering from serious illness in the past two years | g16 | 0=0; 1=1; >1=2 |
| 26 | Number of natural teeth | g21 | 0:5=1; 6:11=0.8; 12:17=0.6; 18:22=0.4; 23:27=0.2; 28:32=0; |
| 27 | Suffering from hypertension | g17a1 | yes=1; no=0 |
| 28 | Suffering from diabetes | g17b1 | yes=1; no=0 |
| 29 | Suffering from heart disease | g17c1 | yes=1; no=0 |
| 30 | Suffering from stroke or cerebrovascular disease | g17d1 | yes=1; no=0 |
| 31 | Suffering from bronchitis, emphysema, pneumonia, asthma | g17e1 | yes=1; no=0 |
| 32 | Suffering from tuberculosis | g17f1 | yes=1; no=0 |
| 33 | Suffering from cataract | g17g1 | yes=1; no=0 |
| 34 | Suffering from Glaucoma | g17h1 | yes=1; no=0 |
| 35 | Suffering from cancer | g17i1 | yes=1; no=0 |
| 36 | Suffering from gastric or duodenal ulcer | g17k1 | yes=1; no=0 |
| 37 | Suffering from Parkinson’s disease | g17l1 | yes=1; no=0 |
| 38 | Suffering from bedsores | g17m1 | yes=1; no=0 |

**Supplementary Table 2.** Inventory of Components Comprising the Mini-Mental State Examination (MMSE)

|  | Variables | Question No. | Values |
| --- | --- | --- | --- |
| 1 | what time of day is it right now? | c11 | wrong=0; not able to answer=0; correct=2 |
| 2 | what month is it right now? | c12 | wrong=0; not able to answer=0; correct=2 |
| 3 | what is the date of the mid-autumn festival? | c13 | wrong=0; not able to answer=0; correct=2 |
| 4 | what is the season right now? | c14 | wrong=0; not able to answer=0; correct=2 |
| 5 | what is the name of this county or district? | c15 | wrong=0; not able to answer=0; correct=2 |
| 6 | repeat the name of "table" at the first time | c21a | wrong=0; not able to answer=0; correct=1 |
| 7 | repeat the name of "apple" at the first time | c21b | wrong=0; not able to answer=0; correct=1 |
| 8 | repeat the name of "clothes" at the first time | c21c | wrong=0; not able to answer=0; correct=1 |
| 9 | $20-$3=? | c31a | wrong=0; not able to answer=0; correct=1 |
| 10 | $20-$3-$3=? | c31b | wrong=0; not able to answer=0; correct=1 |
| 11 | $20-$3-$3-$3=? | c31c | wrong=0; not able to answer=0; correct=1 |
| 12 | $20-$3-$3-$3-$3=? | c31d | wrong=0; not able to answer=0; correct=1 |
| 13 | $20-$3-$3-$3-$3-$3=? | c31e | wrong=0; not able to answer=0; correct=1 |
| 14 | repeat the name of "table" a while later | c41a | wrong=0; not able to answer=0; correct=1 |
| 15 | repeat the name of "apple" a while later | c41b | wrong=0; not able to answer=0; correct=1 |
| 16 | repeat the name of "clothes" a while later | c41c | wrong=0; not able to answer=0; correct=1 |
| 17 | naming "pen" | c51a | wrong=0; not able to answer=0; correct=1 |
| 18 | naming "watch" | c51b | wrong=0; not able to answer=0; correct=1 |
| 19 | repeat a sentence | c52 | wrong=0; not able to answer=0; correct=1 |
| 20 | taking paper using right hand | c53a | wrong=0; unable to do=0; correct=1 |
| 21 | folding paper | c53b | wrong=0; unable to do=0; correct=1 |
| 22 | put paper on the floor | c53c | wrong=0; unable to do=0; correct=1 |
| 23 | draw the figure following the sample | c32 | wrong=0; unable to do=0; correct=1 |
| 24 | # of kinds of food named in one minute | c16 | values >=mean, 2; 0<values<mean, 1; values=0 or not able to answer,0. |

**Supplementary Table 3.** Results of Hypothesis Tests

| Test Type | Comparison (Variables) | Test Statistic | Degrees of Freedom | p-value |
| --- | --- | --- | --- | --- |
| Chi-Square Test | Childhood SES vs. Gender | 45.766 | 2 | 1.153e-10 |
| ANOVA | Childhood SES vs. Age | F = 26.32 | 1, 37262 | 2.92e-07 |
| Chi-Square Test | Childhood SES vs. State | 83.638 | 2 | <2.2e-16 |
| Chi-Square Test | Adulthood SES vs. Gender | 4145.3 | 2 | <2.2e-16 |
| ANOVA | Adulthood SES vs. Age | F = 2300 | 1, 37262 | <2e-16 |
| Chi-Square Test | Adulthood SES vs. State | 1103.3 | 2 | <2.2e-16 |
| Chi-Square Test | Life Course SES vs. Gender | 4639.8 | 8 | <2.2e-16 |
| ANOVA | Life Course SES vs. Age | F = 333.2 | 1, 37262 | < 2e-16 |
| Chi-Square Test | Life Course SES vs. State | 1149.8 | 8 | <2.2e-16 |

**Supplementary Table 4.** Childhood SES-Stratified Estimated Robust, Frailty, and Total Life Expectancy (LE) at Ages 65 and 75, Including 95% Confidence Intervals (CLHLS 1998-2018) After Removing Missing Values

| Childhood SES | Total LE | Robust LE | Frailty LE | Proportion Robust | Proportion Frailty |
| --- | --- | --- | --- | --- | --- |
| Men | | | | |  |
| Age 65 | | | | |  |
| Low childhood SES | 14.31 [3.81, 14.49] | 4.71 [0.73, 4.85] | 9.60 [3.07, 9.75] | 32.91% | 67.09% |
| Medium childhood SES | 14.57 [1.82, 14.76] | 5.09 [0.01, 5.24] | 9.48 [1.80, 9.65] | 34.93% | 65.07% |
| High childhood SES | 14.85 [1.77, 15.16] | 5.49 [0.00, 5.31] | 9.36 [1.77, 9.57] | 36.97% | 63.03% |
| Age 75 | | | | |  |
| Low childhood SES | 9.62 [7.76, 9.76] | 3.03 [2.49, 3.12] | 6.60 [5.31, 6.70] | 31.50% | 68.50% |
| Medium childhood SES | 9.84 [0.01, 9.87] | 3.32 [0.00, 3.41] | 6.52 [0.01, 6.63] | 33.74% | 66.26% |
| High childhood SES | 10.08 [0.00, 10.26] | 3.64 [0.00, 3.77] | 6.44 [0.00, 6.58] | 36.11% | 63.89% |
| Women | | | | |  |
| Age 65 | | | | |  |
| Low childhood SES | 15.54 [12.73, 15.76] | 4.17 [3.37, 4.31] | 11.37 [9.39, 11.57] | 26.83% | 73.17% |
| Medium childhood SES | 15.75 [2.57, 15.97] | 4.50 [0.12, 4.66] | 11.25 [2.47, 11.44] | 28.57% | 71.43% |
| High childhood SES | 15.98 [2.16, 16.25] | 4.88 [0.00, 5.07] | 11.11 [2.16, 11.36] | 30.54% | 69.46% |
| Age 75 | | | | |  |
| Low childhood SES | 10.61 [6.46, 10.76] | 2.72 [1.69, 2.81] | 7.89 [4.77, 8.01] | 25.64% | 74.36% |
| Medium childhood SES | 10.79 [7.17, 10.93] | 2.99 [2.03, 3.08] | 7.81 [5.14, 7.93] | 27.71% | 72.29% |
| High childhood SES | 11.00 [0.00, 11.20] | 3.29 [0.00, 3.41] | 7.71 [0.00, 7.88] | 29.91% | 70.09% |

①SES: socioeconomic status; ②LE: life expectancy; ③Proportion Robust: robust life expectancy accounted for total life expectancy; ④Proportion Frailty: frailty life expectancy accounted for total life expectancy.

**Supplementary Table 5.** Estimated Robust, Frailty, and Total Life Expectancy (LE) at Ages 65 and 75 by Life Course SES Trajectory, Including 95% Confidence Intervals (CLHLS 1998-2018) After Removing Missing Values

| Life Course SES Trajectory (child–adult) | Total LE | Robust LE | Frailty LE | Proportion Robust | Proportion Frailty |
| --- | --- | --- | --- | --- | --- |
| Men | | | | |  |
| Age65 | | | | |  |
| Low–low | 14.055[9.807, 14.264] | 4.418[2.872, 4.562] | 9.637[6.899, 9.827] | 31.43% | 68.57% |
| Low–medium | 14.143[7.852, 14.353] | 4.499[2.154, 4.626] | 9.645[5.695, 9.818] | 31.81% | 68.20% |
| Low–high | 14.232[4.169, 14.431] | 4.576[0.705, 4.721] | 9.656[3.447, 9.805] | 32.15% | 67.85% |
| Medium–low | 14.325[8.873, 14.526] | 4.656[2.555, 4.790] | 9.669[6.318, 9.831] | 32.50% | 67.50% |
| Medium–medium | 14.416[3.322, 14.601] | 4.759[0.382, 4.919] | 9.657[2.937, 9.813] | 33.01% | 66.99% |
| Medium–high | 14.518[2.653, 14.700] | 4.856[0.052, 5.032] | 9.663[2.567, 9.828] | 33.45% | 66.56% |
| High–low | 14.622[2.550, 14.876] | 4.954[0.009, 5.173] | 9.667[2.529, 9.838] | 33.88% | 66.11% |
| High–medium | 14.723[2.694,14.981] | 5.053[0.015, 5.307] | 9.670[2.652, 9.873] | 34.32% | 65.68% |
| High–high | 14.830[2.645, 15.117] | 5.162[0.001, 5.465] | 9.668[2.630, 9.916] | 34.81% | 65.19% |
| Age75 | | | | |  |
| Low–low | 9.482[7.431, 9.626] | 2.901[2.288, 2.998] | 6.581[5.125, 6.697] | 30.59% | 69.41% |
| Low–medium | 9.579[8.154, 9.721] | 3.003[2.599, 3.099] | 6.576[5.548, 6.701] | 31.35% | 68.65% |
| Low–high | 9.678[7.894, 9.817] | 3.106[2.606, 3.188] | 6.572[5.322, 6.685] | 32.09% | 67.91% |
| Medium–low | 9.795[9.078, 9.906] | 3.230[2.984, 3.319] | 6.565[6.009, 6.659] | 32.98% | 67.02% |
| Medium–medium | 9.883[4.018, 10.015] | 3.328[1.358, 3.423] | 6.555[2.660, 6.667] | 33.67% | 66.33% |
| Medium–high | 9.993[0.196, 10.149] | 3.446[0.047, 3.555] | 6.546[0.149, 6.667] | 34.48% | 65.51% |
| High–low | 10.103[0.008, 10.256] | 3.566[0.000, 3.686] | 6.537[0.008, 6.673] | 35.30% | 64.70% |
| High–medium | 10.214[0.070, 10.375] | 3.684[0.009, 3.839] | 6.530[0.061, 6.677] | 36.07% | 63.93% |
| High–high | 10.327[0.002, 10.512] | 3.807[0.000, 3.981] | 6.519[0.002, 6.671] | 36.86% | 63.13% |
| Women | | | | |  |
| Age65 | | | | |  |
| Low–low | 15.364[13.914, 15.614] | 3.956[3.538, 4.097] | 11.408[10.302, 11.651] | 25.75% | 74.25% |
| Low–medium | 15.449[14.846, 15.665] | 4.028[3.815, 4.169] | 11.422[10.985, 11.622] | 26.07% | 73.93% |
| Low–high | 15.525[13.653, 15.740] | 4.103[3.534, 4.242] | 11.422[10.107, 11.608] | 26.43% | 73.57% |
| Medium–low | 15.609[14.103, 15.846] | 4.168[3.695, 4.336] | 11.442[10.403, 11.641] | 26.70% | 73.30% |
| Medium–medium | 15.689[6.634, 15.909] | 4.251[1.203, 4.433] | 11.438[5.431, 11.643] | 27.10% | 72.90% |
| Medium–high | 15.781[4.316, 16.054] | 4.334[0.426, 4.504] | 11.447[3.840, 11.649] | 27.46% | 72.54% |
| High–low | 15.868[3.356, 16.141] | 4.413[0.083, 4.626] | 11.455[3.270, 11.677] | 27.81% | 72.19% |
| High–medium | 15.970[3.247, 16.272] | 4.505[0.010, 4.723] | 11.465[3.203, 11.746] | 28.21% | 71.79% |
| High–high | 16.075[3.393, 16.436] | 4.588[0.020,4.903] | 11.487[3.334, 11.807] | 28.54% | 71.46% |
| Age75 | | | | |  |
| Low–low | 10.520[7.223, 10.680] | 2.646[1.842, 2.748] | 7.874[5.385, 8.008] | 25.15% | 74.85% |
| Low–medium | 10.606[10.365, 10.738] | 2.732[2.622, 2.819] | 7.874[7.683, 7.995] | 25.76% | 74.24% |
| Low–high | 10.702[10.391, 10.836] | 2.840[2.734, 2.923] | 7.863[7.612, 7.983] | 26.54% | 73.47% |
| Medium–low | 10.794[10.568, 10.937] | 2.932[2.820, 3.026] | 7.861[7.656, 7.981] | 27.16% | 72.83% |
| Medium–medium | 10.891[10.122, 11.044] | 3.044[2.834, 3.138] | 7.847[7.277, 7.979] | 27.95% | 72.05% |
| Medium–high | 10.993[5.794, 11.157] | 3.153[1.695, 3.266] | 7.841[4.099, 7.988] | 28.68% | 71.33% |
| High–low | 11.092[1.216, 11.260] | 3.259[0.353, 3.392] | 7.833[0.864, 7.993] | 29.38% | 70.62% |
| High–medium | 11.198[0.021, 11.398] | 3.372[0.000, 3.519] | 7.826[0.020, 8.029] | 30.11% | 69.89% |
| High–high | 11.305[1.174, 11.552] | 3.481[0.365, 3.669] | 7.824[0.812, 8.042] | 30.79% | 69.21% |

①SES: socioeconomic status; ②LE: life expectancy; ③Proportion Robust: robust life expectancy accounted for total life expectancy; ④Proportion Frailty: frailty life expectancy accounted for total life expectancy.

# Estimation methods of life expectancy (LE)

In this research, we utilized the *elect* (1)and *msm* (2) packages in R to estimate LE for specific states using a continuous-time multi-state model. This approach enables the calculation of LE across various states and is not confined to progressive processes.

As illustrated in Supplementary Figure 2, the multi-state model comprises two living states (robust: state 1, frailty: state 2) and one absorbing state (death: state 3). Due to the dynamic characteristics of frailty, the model allows for transitions (indicated by arrows) between any living state and any other state, including remaining in the same state or transitioning to the death state (3).

Consider a finite state space represented by {1,2,…,D}, where D signifies the death state. Let *Y*_t_ represent the state at age *t*, and let *x* denote the vector of covariate values that remain constant over time.

The LE in a living state *s*, given that the individual is in state *r* at age *t*, for $r,s\in\left\{ 1,2,\ldots,D-1 \right\}$*,*is defined as follows:

$e_{rs}\left( t|x \right)=\int_{0}^{\infty} \mathbb{P(}Y_{t+u}=s|Y_{t}=r,x)du$  (1)

Here, $\mathbb{P}\left( Y_{t+u}=s | Y_{t}=r,x \right)$represents the probability of transitioning to state *s* at age *t+u*, given that the individual was in state *r* at age *t* with covariate values *x*. The marginal LE in state *s*, regardless of the initial state at age *t*, is defined as:

$e_{\cdot s}\left( t | x \right)=\sum_{r\neq D} \mathbb{P}\left( Y_{t}=r | x \right)e_{rs}(t|x)$ (2)

Here, $\mathbb{P}\left( Y_{t}=r | x \right)$denotes the probability of being in state *r* at age *t* for $r\in\left\{ 1,2,\ldots,D-1 \right\}.$The total LE at age *t* is defined as:

$e\left( t | x \right)=\sum_{s\neq D} e_{\cdot s}\left( t | x \right)$ (3)

To estimate LE, we use longitudinal data to determine transition probabilities and state distributions. Adopting the same notation as previously, we assume that the data for individual *i* and observation *j* are represented by (*y_ij_, t_ij_, x_i_*), where $i\in\left\{ 1,\ldots,N \right\}$ and $j\in\{1,\ldots,n_{i}\}$ *.*Transition probabilities are derived from a multi-state model, with hazards defined by:

$h_{rs}\left( t_{ij} \right)=exp(\beta_{rs}+\xi_{rs}t_{ij}+\gamma_{rs}x_{i})$ (4)

This model is estimated using the *msm* package.

The state distribution at age *t* is modeled using a multinomial regression approach, defined as follows:

$\mathbb{P}\left( Y_{t}=r | x \right)=\frac{exp(\eta_{r}(t))}{1+\sum_{r\neq D} exp(\eta_{r}(t))}$ (5)

where $\eta_{r}\left( t \right)=\alpha_{r0}+\alpha_{r1}t+\alpha_{r2}x$

for $r\in\left\{ 1,2,\ldots,D-1 \right\}$. By setting *α_10_* = *α_11_* = *α_12_* = 0, we designate *r*=1 as the reference category. This model is estimated in *elect* using the *multinom* function in the *nnet* package*.*

The LE defined in equations (1), (2), and (3) can be calculated using the *elect* package by applying the parameters estimated from the multi-state model and the multinomial regression model.

# Supplementary Data

The data used in this study are openly available in the Peking University open research data at: <https://opendata.pku.edu.cn/dataset.xhtml?persistentId=doi:10.18170/DVN/XRV2WN>

# References

1. van den Hout A, Sum Chan M, Matthews F. Estimation of life expectancies using continuous-time multi-state models. *Comput Methods Programs Biomed* (2019) 178:11–18. doi: 10.1016/j.cmpb.2019.06.004

2. Jackson C. Multi-State Models for Panel Data: The msm Package for R. *J Stat Softw* (2011) 38:1–28. doi: 10.18637/jss.v038.i08

3. Gao J, Wang Y, Xu J, Jiang J, Yang S, Xiao Q. Life expectancy among older adults with or without frailty in China: multistate modelling of a national longitudinal cohort study. *BMC Med* (2023) 21:101. doi: 10.1186/s12916-023-02825-7
